# Room air constituent concentrations from use of electronic nicotine delivery systems and cigarettes using different ventilation conditions

**DOI:** 10.1038/s41598-021-80963-9

**Published:** 2021-01-18

**Authors:** Michael J. Oldham, Anil Sehgal, Gal Cohen, Joey Chen, Blair Evans, Daniel Heraldez

**Affiliations:** JUUL Labs, Inc., 560 20th Street, San Francisco, USA

**Keywords:** Environmental sciences, Chemistry

## Abstract

To assess potential exposure of non-users to exhaled constituents from pod and cartridge electronic nicotine delivery systems (ENDS) products, an environmental clinical study was conducted with (n = 43) healthy adult smokers. Room air concentrations of 34 selected constituents (nicotine, propylene glycol, glycerin, 15 carbonyls, 12 volatile organic compounds, and 4 trace metals) and particle number concentration (0.3 to 25 µm) were compared from use of two ENDS products and conventional cigarettes using room ventilations representative of a residential, an office or a hospitality setting over a 4-h. exposure period. Products used were JUUL ENDS, Virginia Tobacco flavor (Group I), VUSE Solo, Original flavor (Group II) (5.0 and 4.8% nicotine by weight, respectively) and subjects’ own conventional cigarettes (Group III). Cumulative 4-h room air sampling and particle counting were performed during prescribed (Groups I and II) and ad libitum product use (all Groups). Conventional cigarette use resulted in significantly more constituents detected and higher 4-h cumulative constituent concentrations compared to use of the ENDS products tested, except for the predominant ENDS ingredients, propylene glycol and glycerin. Use of conventional cigarettes also resulted in greater total particle number concentration than either prescribed or ad libitum use of either of the ENDS used in this study.

## Introduction

Electronic nicotine delivery systems (ENDS; also called e-cigarettes) have been gaining popularity in the United States, especially closed system ENDS using pods or cartridges. Concern has been raised about potential exposure of non-users to exhaled constituents from these ENDS^1^. Initial reports of room constituent concentrations were from puffing machine generated ENDS aerosols that assumed 100% percent of what was generated was released into room air^2–5^. These machine-based studies used constant puffing parameters and neglected aerosol deposition in the respiratory tract of the user prior to some fraction being exhaled. From this initial work, two assessment approaches of potential non-user exposure to exhaled constituents from ENDS have evolved. One assessment approach has been to measure air constituent concentrations in locations where ENDS are or have been used. Sampled locations include shops selling ENDS^6–8^ and homes where one or more occupants report using ENDS^8–10^. In some cases, this assessment approach includes measuring biomarkers of exposure in saliva, plasma or urine^11–15^. The second assessment approach has used more controlled situations where ENDS users are confined to a room with prescribed or ad libitum product use^3,4,16–23^ and/or have included exhaled breath analysis^24–28^. Results from both approaches are consistent in that compared to inhaled constituent concentrations, low concentrations of constituents (nicotine, propylene glycol, glycerin and formaldehyde) have been measured in exhaled breath^19,24,26,27^ and in environmental sampling during prescribed or ad libitum ENDS use. Both approaches have shown that exposure of non-users is possible, but most studies have demonstrated this exposure is significantly less than exposure to secondhand smoke from combustible cigarettes ^10,13,19,24,25,28–30^. In large part because unlike cigarettes, which generate sidestream smoke continuously between puffs, there are no emissions from ENDS between puffs. There are also more chemicals and larger amount of chemicals (except for glycerin and propylene glycol, primary E-vapor product ingredients) in the exhaled breath of cigarette smokers compared to ENDS users^24,25,28^, which also contributes to potential exposure of non-users.

Although potential differences have been identified between types of ENDS^19^ (cig-a-like vs. tanks), few if any of these studies have quantified the potential non-product user exposure using popular pod- or cartridge-based ENDS, especially JUUL ENDS. Additionally, when subjects have been confined within a room, all previous studies have utilized only a single room ventilation rate, which has limited extrapolation of the results. Therefore, we conducted an environmental clinical study comparing room air concentrations of selected constituents from smokers using a JUUL ENDS containing 5.0% nicotine by weight, VUSE Solo ENDS containing 4.8% nicotine by weight or conventional cigarettes (participants usual brand) using three different room ventilations rates that are representative of a residential, an office or a hospitality setting. The three different ventilation conditions were anticipated to result in a range of concentrations producing a dose response for the measured constituents because different amounts of fresh air that were used. The different ventilation rates and amounts of fresh air used were consistent with standards by the American Society of Heating, Refrigeration and Air-Conditioning Engineers Inc^31^. for living spaces (Table 1; approximately 30% less for the office and approximately 67% less for the hospitality ventilation conditions compared to the residential ventilation condition).

**Table 1:** American Society of Heating, Refrigeration and Air-Conditioning Engineers Inc. ventilation standards.

Ventilation condition	Total airflow		Fresh air	
	Minimum air exchanges/hr	Minimum flow rate (cubic feet/min)	Minimum air exchanges/h	Minimum flow rate (cubic feet/min)
Residential	4.5	345	1.1	85
Office	7.5	575	1.57	121
Hospitality	15.0	1150	3.3	255

## Results

### Study participants

A total of 43 study participants (25 males and 18 females) were enrolled. The mean age was 43.8 years and ranged from 24 to 63 years. The mean number of reported cigarettes smoked per day was 15.2 with a range of 10–25 cigarettes per day. The mean duration of cigarette smoking reported was 22.4 years with a range of 0.6 to 48 years.

Group I participants consisted of 4 males and 6 females with an average age of 46.2 years who reported using a mean of 13.1 cigarettes per day and had smoked for an average of 22.7 years. Group II participants consisted of 6 males and 4 females with an average age of 42.3 years who reported using a mean of 15.1 cigarettes per day and had smoked for an average of 19.9 years. Group III participants consisted of 7 males and 3 females with an average age of 42.2 years who reported using a mean of 14.4 cigarettes per day and had smoked for an average of 22.5 years.

Three study participants in Group I reported a total of four adverse events (3 of moderate severity) and fourteen adverse events (3 of moderate severity) were reported by six study participants in Group II. No adverse effects were reported from participants in Group III. No adverse events were judged to be serious and related to product use by the principal investigator or resulted in withdrawal from the study. Seventeen adverse events were judged by the principal investigator to be possibly related to product use including: 8 reports of headache; 2 reports of nasal congestion; 2 reports of rhinorrhea; 2 reports of sore/throat irritation; 1 report of viral upper respiratory tract infection, 1 report of flu like symptoms and 1 report of back pain. One study participant from Group I withdrew due to a family emergency. Two study participants from Group II withdrew from the study, one withdrew due to a family emergency and the second participant was discontinued from the office and hospitality conditions due to study procedure non-compliance. All remaining study participants completed the study according to the protocol.

### Product use

The amount of e-liquid used by Groups I and II with prescribed and ad libitum product use is shown in Table 2. For Groups I and II, the average amount of e-liquid used during prescribed product use was relatively consistent between the different ventilation conditions, however Group II consistently used more e-liquid than Group I during prescribed product use. For Group II, the average amount of e-liquid used during ad libitum product use was similar to the amount used during prescribed product use and was also relatively consistent between the different ventilation condition. There were differences in the average amount of e-liquid used between prescribed and ad libitum product use in Group I for the residential and hospitality ventilation conditions, but not the office ventilation condition.

**Table 2:** E-liquid used during prescribed and ad libitum product use for Groups I and II.

Parameter	Residential		Office		Hospitality	
	Prescribed*	Ad libitum	Prescribed*	Ad libitum	Prescribed*	Ad libitum
**Group I E-liquid used (mg)**						
Mean ± SD	136.8 ± 60.2	205.1 ± 119.5	124.2 ± 29.0	137.6 ± 124.1	117.8 ± 55.2	151.4 ± 142.3
Median	124.0	226.5	117.0	127.0	107.5	93.0
Range	65.0–286.0	53.0–385.0	95.0–186.0	22.0–443.0	25.0–183.0	43.0–428
N	10	10	9	9	10	9
**Group II E-liquid used (mg)**						
Mean ± SD	191.9 ± 79.8	189.3 ± 109.6	215.5 ± 107.8	212.4 ± 175.5	192.3 ± 94.6	168.2 ± 195.7
Median	186.0	206.5	237.5	159.0	227.0	84.0
Range	37.0–299.0	10.0–394.0	25.0–364	14.0–573.0	17.0–334.0	13.0–606.0
N	10	10	8	8	9	9

*In addition to the 80 puffs taken during prescribed use, an additional 20 puffs were taken prior to weighing the pods, so values represent 100 puffs.

### Selected constituent concentrations

**Figure 1:**
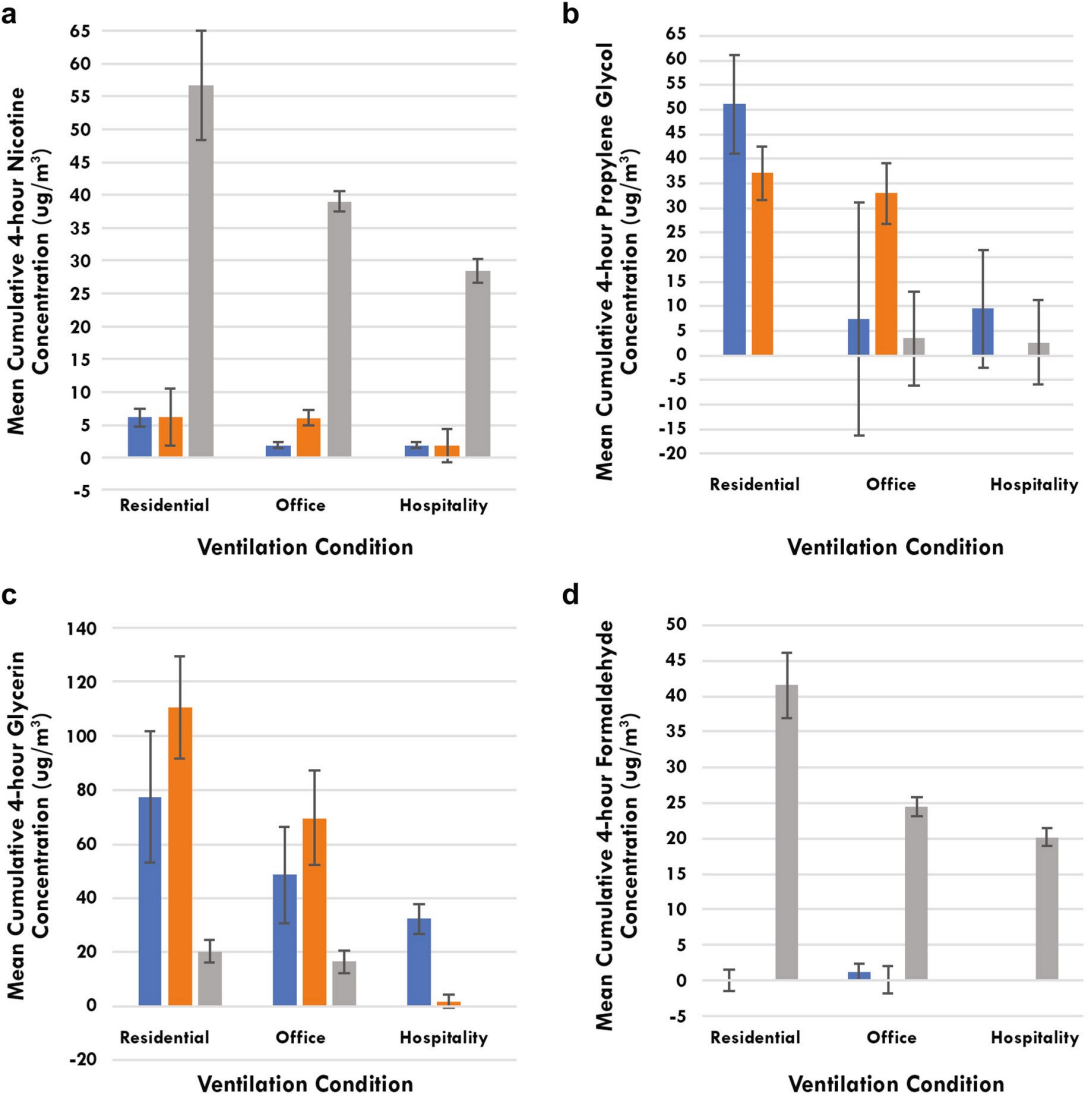
Comparison of mean cumulative (± SD) nicotine, propylene glycol, glycerin and formaldehyde concentrations measured after 4-h of ad libitum product use for all Groups. Group I—Blue bars, Group II—Orange bars, and Group III gray bars. Missing bars indicate that the cumulative mean concentrations were below baseline values.

The mean cumulative 4-h concentration of each constituent that was measured above the limit of quantification for Groups I, II and III are provided in Tables 3, 4, and 5. To determine which of the 34 measured constituents were related to product use, five criteria were used to sort the mean cumulative 4-h constituent concentration data (Table 6). Constituents were removed from consideration if : (1) They were never found above limit of quantification (LOQ); (2) Were not statistically different from baseline concentrations in any Group; (3) Were not statistically different from the baseline concentration measured prior to product use; (4) There was no dose response as a function of amount of fresh air supplied in the different ventilation conditions (i.e., approximately 30% less for office ventilation and approximately 67% less for the hospitality ventilation) for prescribed product use for Groups I and II and ad libitum product use for Group III; and (5) Did not correlate with the difference in amount of e-liquid used (Table 2) between prescribed vs. ad libitum product use. For each ENDS three constituents/Group met all five criteria, nicotine, propylene glycol, and glycerin (Group I) and glycerin, 1,3-butadiene, and isoprene (Group II) (Table 6). In comparison, for Group III there were eleven constituents (nicotine, glycerin, acetaldehyde, acetone, formaldehyde, propionaldehyde, 1,3-butadiene, benzene, ethylbenzene, furan, and isoprene) that met all five criteria, (Table 6). For Group III, not only were there significantly more constituents that met the five criteria, but also their measured concentrations were significantly greater, except for the main ENDS ingredients, propylene glycol and glycerin (Tables 3, 4, 5). For example, the mean 4-h cumulative nicotine and formaldehyde concentrations from ad libitum use of ENDS were over 84% and 95% less than the nicotine and formaldehyde concentrations from ad libitum use of conventional cigarettes, respectively, when comparing within ventilation conditions (Fig. 1). For the main ENDS ingredients, propylene glycol and glycerin, ad libitum use of ENDS resulted in concentrations that were several fold higher than concentrations measured from ad libitum use of conventional cigarettes (Fig. 1).

**Table 3:** Mean (± SD; N = 4) cumulative (4-h) constituent concentrations changes from baseline values for Group I for any constituent detected above LOQ for at least one puffing regimen for any group.

Constituent	LOQ (µg/m^3^)	Group I					
		Residential (µg/m^3^)		Office (µg/m^3^)		Hospitality (µg/m^3^)	
		Prescribed	Ad libitum	Prescribed	Ad libitum	Prescribed	Ad libitum
Nicotine*	1.87	**4.01 ± 1.63**	**6.14 ± 1.31**	**1.37 ± 0.37**	**1.95 ± 0.46**	**0.75 ± 0.20**	**1.91 ± 0.42**
Propylene Glycol	31.93	**38.54 ± 14.05**	**51.17 ± 10.03**	8.52 ± 21.71	7.45 ± 23.66	5.88 ± 11.03	9.51 ± 11.94
Glycerin	33.66	**70.85 ± 27.95**	**77.50 ± 24.25**	**33.12 ± 13.76**	**48.60 ± 17.82**	**17.78 ± 5.06**	**32.30 ± 5.41**
Acetaldehyde	24.82	BBV	0.52 ± 2.97	**0.60 ± 0.18**	BBV	0.14 ± 0.31	BBV
Acetone*	24.71	2.78 ± 2.61	BBV	**10.85 ± 4.17**	5.10 ± 6.53	**6.40 ± 2.58**	6.55 ± 5.31
Acrolein	24.82	BBV	BBV	BBV	BBV	0.05 ± 0.17	0.55 ± 0.64
Benzaldehyde	28.82	BBV	BBV	BBV	BBV	0.05 ± 0.19	0.63 ± 0.74
Butyraldehyde	25.03	BBV	BBV	BBV	BBV	0.09 ± 0.31	1.09 ± 1.27
Crotonaldehyde	25.03	BBV	BBV	BBV	BBV	0.03 ± 0.09	0.31 ± 0.36
Formaldehyde	24.71	0.05 ± 2.10	0.03 ± 1.45	**1.65 ± 0.24**	1.13 ± 1.18	BBV	BBV
Hexanaldehyde	24.82	BBV	BBV	BBV	BBV	0.07 ± 0.27	0.78 ± 0.94
Isovaleraldehyde	24.82	BBV	BBV	BBV	BBV	0.04 ± 0.13	0.43 ± 0.50
Methyl ethyl ketone	24.189	BBV	0.93 ± 2.30	BBV	BBV	BBV	BBV
m&p tolualdehyde	49.64	BBV	BBV	BBV	BBV	0.04 ± 0.14	0.45 ± 0.53
o-Tolualdehyde	25.03	BBV	BBV	BBV	BBV	0.04 ± 0.15	0.49 ± 0.57
Propionaldehyde	24.82	1.47 ± 3.00	**5.11 ± 1.65**	0.82 ± 0.94	BBV	0.03 ± 0.43	0.82 ± 0.84
Valeraldehyde	24.82	BBV	BBV	BBV	BBV	0.04 ± 0.12	0.40 ± 0.46
2,5-Dimethylbenzaldehyde	25.03	BBV	BBV	BBV	BBV	0.11 ± 0.42	1.35 ± 1.58
1,3-Butadiene*	0.3	BBV	BBV	0.00 ± 0.08	0.03 ± 0.05	0.03 ± 0.05	0.03 ± 0.05
2-Nitropropane	0.7	0.0 ± 0.0	0.0 ± 0.0	0.0 ± 0.0	0.0 ± 0.0	0.0 ± 0.0	0.0 ± 0.0
Benzene*	0.3	**0.25 ± 0.06**	**0.35 ± 0.06**	0.13 ± 0.13	**0.35 ± 0.06**	BBV	**0.28 ± 0.15**
Ethylbenzene*	0.3	0.15 ± 0.13	0.05 ± 0.06	**0.60 ± 0.22**	**0.25 ± 0.06**	BBV	**0.48 ± 0.05**
Ethylene oxide	3.4	0.00 ± 0.08	0.03 ± 0.10	0.03 ± 0.05	BBV	0.05 ± 0.06	0.03 ± 0.05
Furan	0.7	**1.40 ± 0.14**	**0.90 ± 0.56**	0.0 ± 0.0	0.0 ± 0.0	0.0 ± 0.0	0.0 ± 0.0
Isoprene*	0.3	0.55 ± 0.42	BBV	0.05 ± 1.11	BBV	BBV	0.73 ± 0.51
Nitromethane	0.7	0.0 ± 0.0	0.0 ± 0.0	0.0 ± 0.0	0.0 ± 0.0	0.0 ± 0.0	0.0 ± 0.0
Propylene oxide	3.4	0.00 ± 0.08	0.03 ± 0.10	0.03 ± 0.05	BBV	0.05 ± 0.06	0.03 ± 0.05
Toluene*	0.3	**0.95 ± 0.13**	**0.58 ± 0.05**	**0.65 ± 0.29**	**1.13 ± 0.05**	BBV	**1.13 ± 0.05**
Vinyl acetate	0.7	0.0 ± 0.0	0.0 ± 0.0	0.0 ± 0.0	0.0 ± 0.0	0.0 ± 0.0	0.0 ± 0.0

Statistically significant constituent values from baseline (p < 0.05) are in bold. Statistically significant differences (p < 0.05) found in baseline concentrations (N = 4) between ventilation conditions are denoted with an asterisk.

*BBV* Below baseline value, *LOQ* Limit of Quantification is based upon the amount of air sampled and is a composite average from all 4 air samplers used in the six runs.

**Table 4:** Mean (± SD; N = 4) cumulative (4-h) constituent concentrations changes from baseline values for Group II for any constituent detected above LOQ for at least one puffing regimen for any group.

Constituent	LOQ (µg/m^3^)	Group II					
		Residential (µg/m^3^)		Office (µg/m^3^)		Hospitality (µg/m^3^)	
		Prescribed	Ad libitum	Prescribed	Ad libitum	Prescribed	Ad libitum
Nicotine*	1.87	3.45 ± 3.95	6.23 ± 4.31	**5.77 ± 1.64**	**6.11 ± 1.12**	2.77 ± 2.75	1.87 ± 2.56
Propylene glycol	31.93	**30.34 ± 5.08**	**37.12 ± 5.40**	**36.13 ± 5.49**	**32.95 ± 6.12**	0.25 ± 28.01	BBV
Glycerin	33.66	**81.80 ± 4.58**	**110.55 ± 18.82**	**69.20 ± 23.88**	**69.63 ± 17.33**	2.77 ± 2.75	1.87 ± 2.56
Acetaldehyde	24.82	BBV	0.40 ± 1.10	0.13 ± 1.09	BBV	**0.68 ± 0.27**	0.01 ± 0.25
Acetone*	24.71	2.53 ± 5.97	BBV	**9.75 ± 6.05**	3.28 ± 12.49	3.20 ± 5.62	BBV
Acrolein	24.82	0.04 ± 0.19	BBV	0.06 ± 0.13	BBV	BBV	0.03 ± 0.04
Benzaldehyde	28.82	0.05 ± 0.22	0.00 ± 0.24	0.06 ± 0.15	BBV	BBV	0.03 ± 0.07
Butyraldehyde	25.03	0.08 ± 0.36	BBV	0.10 ± 0.26	BBV	BBV	0.06 ± 0.06
Crotonaldehyde	25.03	0.02 ± 0.11	0.00 ± 0.12	0.03 ± 0.07	BBV	BBV	0.02 ± 0.02
Formaldehyde	24.71	BBV	BBV	BBV	0.13 ± 1.88	0.09 ± 0.33	BBV
Hexanaldehyde	24.82	0.08 ± 0.26	0.03 ± 0.31	0.09 ± 0.20	BBV	BBV	0.05 ± 0.06
Isovaleraldehyde	24.82	0.03 ± 0.15	BBV	0.04 ± 0.10	BBV	BBV	0.02 ± 0.03
Methyl ethyl ketone	24.189	BBV	0.41 ± 0.95	BBV	0.33 ± 0.80	BBV	BBV
m&p tolualdehyde	49.64	0.04 ± 0.16	BBV	0.05 ± 0.11	BBV	BBV	0.03 ± 0.03
o-Tolualdehyde	25.03	0.04 ± 0.17	BBV	0.05 ± 0.11	BBV	BBV	0.03 ± 0.03
Propionaldehyde	24.82	**12.89 ± 3.64**	BBV	**62.09 ± 9.13**	0.39 ± 0.72	BBV	BBV
Valeraldehyde	24.82	0.03 ± 0.14	BBV	0.04 ± 0.09	BBV	BBV	0.02 ± 0.03
2,5-Dimethylbenzaldehyde	25.03	0.11 ± 0.47	0.00 ± 0.52	0.15 ± 0.30	BBV	BBV	0.09 ± 0.08
1,3-Butadiene	0.3	**0.20 ± 0.08**	**0.15 ± 0.06**	0.05 ± 0.06	BBV	0.0 ± 0.0	0.0 ± 0.0
2-Nitropropane	0.7	0.03 ± 0.05	0.0 ± 0.0	0.0 ± 0.0	0.0 ± 0.0	0.0 ± 0.0	0.0 ± 0.0
Benzene	0.3	0.05 ± 0.06	0.03 ± 0.05	0.08 ± 0.10	0.03 ± 0.05	0.00 ± 0.14	0.05 ± 0.17
Ethylbenzene*	0.3	BBV	BBV	0.05 ± 0.10	0.0 ± 0.0	**0.48 ± 0.21**	BBV
Ethylene oxide	3.4	0.15 ± 0.24	0.00 ± 0.08	0.05 ± 0.06	0.00 ± 0.08	0.03 ± 0.05	0.0 ± 0.0
Furan	0.7	0.03 ± 0.05	0.0 ± 0.0	0.0 ± 0.0	0.0 ± 0.0	0.0 ± 0.0	0.0 ± 0.0
Isoprene*	0.3	**1.10 ± 0.68**	BBV	0.13 ± 0.99	0.65 ± 0.59	BBV	BBV
Nitromethane	0.7	BBV	BBV	0.0 ± 0.0	0.0 ± 0.0	0.0 ± 0.0	0.0 ± 0.0
Propylene oxide	3.4	0.15 ± 0.24	0.00 ± 0.08	0.05 ± 0.06	0.00 ± 0.08	0.03 ± 0.05	0.0 ± 0.0
Toluene*	0.3	BBV	BBV	**0.13 ± 0.05**	**0.58 ± 0.10**	**2.43 ± 0.22**	BBV
Vinyl acetate	0.7	0.03 ± 0.05	0.0 ± 0.0	0.0 ± 0.0	0.0 ± 0.0	0.0 ± 0.0	0.0 ± 0.0

Statistically significant constituent values from baseline (p < 0.05) are in bold. Statistically significant differences (p < 0.05) found in baseline concentrations (N = 4) between ventilation conditions are denoted with an asterisk.

*BBV* Below baseline value, *LOQ* Limit of Quantification is based upon the amount of air sampled and is a composite average from all 4 air samplers used in the six runs.

**Table 5:** Mean (± SD; N = 4) cumulative (4-h) constituent concentrations changes from baseline values for Group III for any constituent detected above LOQ for at least one puffing regimen for any group.

Constituent	LOQ (µg/m^3^)	Group III Ad libitum (µg/m^3^)		
		Residential	Office	Hospitality
Nicotine*	1.87	**56.68 ± 8.28**	**39.02 ± 1.59**	**28.49 ± 1.76**
Propylene glycol*	31.93	BBV	3.43 ± 9.62	2.63 ± 8.62
Glycerin*	33.66	**20.38 ± 4.05**	**16.60 ± 4.22**	BBV
Acetaldehyde	24.82	**58.45 ± 8.87**	**37.28 ± 2.26**	**28.98 ± 1.70**
Acetone*	24.71	**21.43 ± 4.97**	6.83 ± 11.12	5.13 ± 7.35
Acrolein	24.82	**0.32 ± 0.18**	0.03 ± 0.03	**0.10 ± 0.05**
Benzaldehyde	28.82	**0.38 ± 0.23**	BBV	**0.11 ± 0.05**
Butyraldehyde	25.03	**0.65 ± 0.38**	BBV	**0.18 ± 0.08**
Crotonaldehyde	25.03	**0.18 ± 0.10**	BBV	**0.05 ± 0.03**
Formaldehyde	24.71	**41.53 ± 4.61**	**24.48 ± 1.39**	**20.16 ± 1.27**
Hexanaldehyde	24.82	**0.46 ± 0.26**	BBV	0.13 ± 0.09
Isovaleraldehyde	24.82	**0.26 ± 0.14**	BBV	**0.07 ± 0.04**
Methyl ethyl ketone	24.189	2.79 ± 1.84	**3.72 ± 0.66**	3.36 ± 2.33
m&p tolualdehyde	49.64	**0.27 ± 0.15**	BBV	**0.08 ± 0.04**
o-Tolualdehyde	25.03	**0.29 ± 0.16**	BBV	**0.08 ± 0.04**
Propionaldehyde	24.82	**1.25 ± 0.55**	0.84 ± 2.41	BBV
Valeraldehyde	24.82	**0.24 ± 0.13**	BBV	**0.07 ± 0.04**
2,5-Dimethylbenzaldehyde	25.03	**0.80 ± 0.43**	BBV	**0.24 ± 0.13**
1,3-Butadiene*	0.3	**9.58 ± 0.78**	**4.85 ± 0.33**	**3.98 ± 1.01**
2-Nitropropane	0.7	BBV	0.0 ± 0.0	0.0 ± 0.0
Benzene*	0.3	**8.38 ± 0.67**	**3.73 ± 0.39**	**2.95 ± 0.17**
Ethylbenzene*	0.3	**3.10 ± 0.29**	**1.15 ± 0.06**	**1.05 ± 0.06**
Ethylene oxide	3.4	BBV	0.0 ± 0.0	0.00 ± 0.08
Furan*	0.7	**6.30 ± 1.87**	**4.93 ± 0.28**	**3.50 ± 0.54**
Isoprene*	0.3	**55.63 ± 5.27**	**30.13 ± 4.04**	**25.08 ± 4.86**
Nitromethane	0.7	BBV	0.0 ± 0.0	0.0 ± 0.0
Propylene oxide	3.4	BBV	0.0 ± 0.0	0.0 ± 0.08
Toluene*	0.3	**13.75 ± 1.35**	**5.10 ± 0.27**	**5.45 ± 0.48**
Vinyl acetate	0.7	BBV	0.0 ± 0.0	0.0 ± 0.0

Statistically significant constituent values from baseline (p < 0.05) are in bold. Statistically significant differences (p < 0.05) found in baseline concentrations (N = 4) between ventilation conditions are denoted with an asterisk.

*BBV* Below baseline value, *LOQ* Limit of Quantification is based upon the amount of air sampled and is a composite average from all 4 air samplers used in the three runs.

**Table 6:**
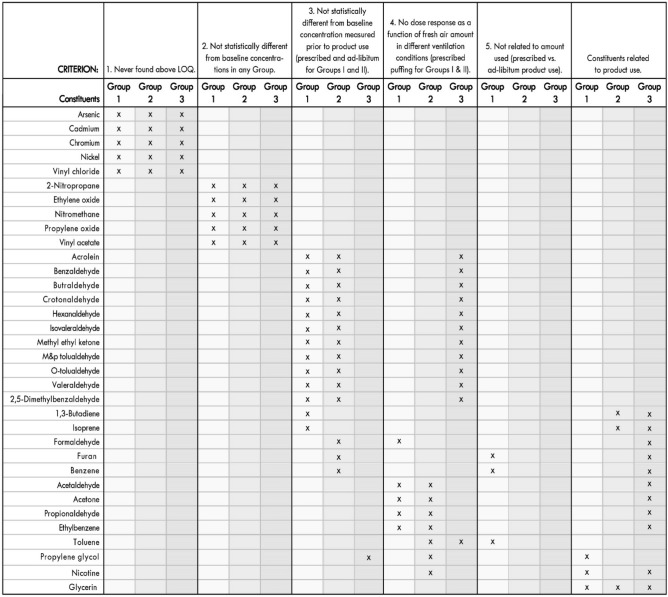
Criteria used for deciding which measured constituents are a function of product use.

The 4-h cumulative concentrations of constituents during ad libitum use were generally higher than during prescribed use in Groups I and II. There were also 4-h cumulative concentrations of one constituent in Group I (acetone) and two in Group II (ethylbenzene and toluene) that increased as a function of the amount of fresh air dilution (Tables 3, 4).

Baseline measurements were performed with the participants in the exposure chamber without any product use on Day 1 for each ventilation condition. For some constituents, there was substantial variability in baseline concentrations measured between ventilation conditions within a group (Tables 3, 4, 5) and between groups (Supplemental Table S4). Comparing mean baseline constituent concentrations, measured from all three groups and ventilation conditions (N = 12), to baseline concentrations obtained prior to product use in the residential ventilation condition (N = 4), resulted in statistically significant differences (p < 0.05) in 11 of the 34 constituent concentrations (nicotine, propylene glycol, glycerin, acetone, 1,3-butadiene, benzene, ethyl benzene, furan, isoprene, nickel, and toluene). Performing the same comparison for baseline measurements obtained prior to the office and hospitality ventilation conditions resulted in statistically significant differences (p < 0.05) for 7 of the 34 constituent concentrations (nicotine, acetone, 1,3-butadiene, benzene, ethyl benzene, nickel, and toluene) and 3 of the constituent concentrations (acetone, ethyl benzene, and toluene), respectively. Reduction of the number of significant differences in baseline constituent concentrations as a function of increasing fresh air dilution, suggests that these constituents were from participants and not increased amounts of high-efficiency particulate air (HEPA) filter fresh air. Since the different ventilation conditions within and between Groups were conducted on different days, another potential source of variability are daily variations in background concentrations of constituents. Background carbonyl measurements were performed without participants in the exposure chamber prior to and after baseline measurements, prescribed and ad libitum product use. Differences in background concentrations (> 20% between measurements) were found between ventilation conditions within a group and between groups for acetaldehyde, acetone, and propionaldehyde (Supplemental Figs. S1–S45).

### Particle concentration

**Figure 2:**
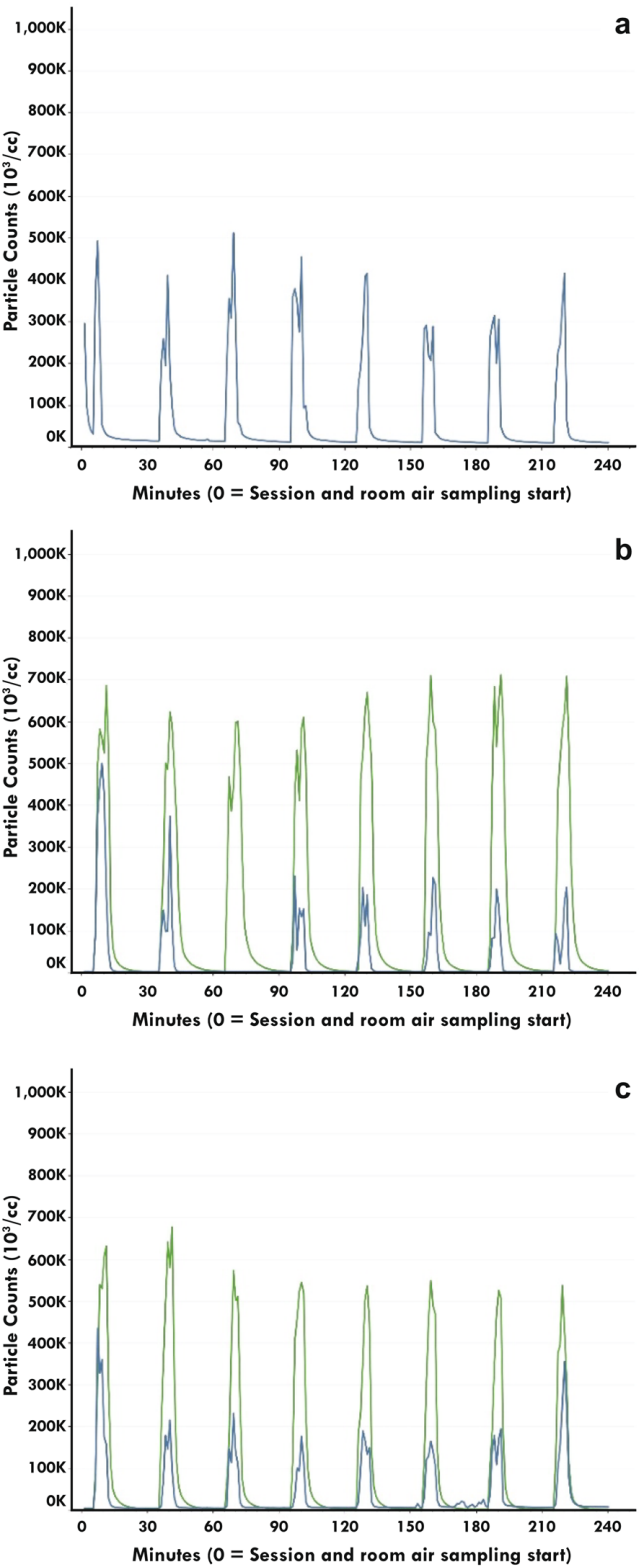
Comparison of the particle number concentration measured from prescribed product use in Groups I (blue line) and II (green line) in each ventilation condition (A = residential; B = Office; C = Hospitality). Note that for Group II in the residential ventilation condition there was no particle number concentration due incorrect device setup.

**Figure 3:**
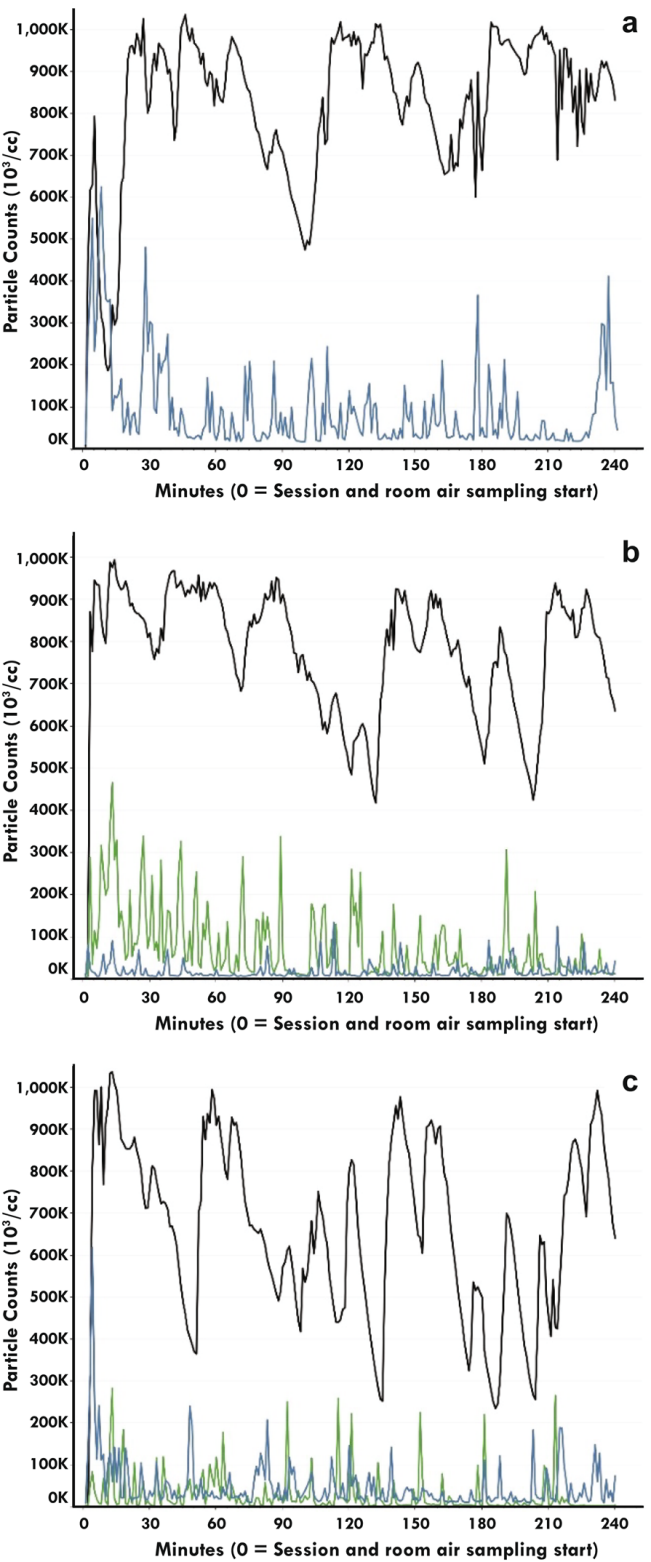
Comparison of the particle number concentration measured from ad libitum product use in Groups I, II and III (blue, green and black lines respectively) in each ventilation condition (A = residential; B = Office; C = Hospitality).

Temporally, the particle number concentration for Groups I and II mirrored the prescribed puffing regimen for each ventilation condition (Fig. 2). Particle number concentration for the residential condition with Group II were not obtained (Fig. 2A) because the particle sampler was set up incorrectly. An instrument error also occurred for four minutes in the office ventilation condition for Group I (Fig. 2B). In the office and hospitality ventilation condition, total particle number concentration during product use were less for Group I compared with Group II (Fig. 2B,C). Total particle number concentration under ad libitum conditions were generally lower than those from the prescribed puffing regimen for Groups I and II, however there were instances when the total particle number concentrations were roughly equivalent (Figs. 2 and 3). Total particle number concentration during ad libitum product use were substantially higher for Group III compared to Groups I and II in the residential (Fig. 3A) and office (Fig. 3B) ventilation conditions. In the hospitality ventilation condition, there was evidence of reduced total particle number concentration that were most prominent in Group III with some overlap in total particle number concentration with Groups I and II (Fig. 3C).

## Discussion

The use of multiple ventilation conditions enabled use of a dose response (Criteria #4) to help determine which of the measured constituents were correlated to product use. For ENDS used in Groups I and II only three of the 34 constituents measured were correlated to product use while eleven of the 34 constituents were correlated to use of conventional cigarettes (met all five criteria). Cumulative concentration of constituents after ENDS and conventional cigarette use were consistent with previously published environmental studies^3,16,18,19^. Differences in specific constituents detected and their cumulative concentration between our results and the previous environmental studies, are not only due to the different ENDS tested, different puffing regimens, different subject populations, but also different room sizes and ventilation rates used in the current study. For example, one study^16^ placed nine occasional smokers (< 10 cigarettes/week) in a smaller room (18 m^2^ & 45 m^3^) with 0.56 air exchanges/hour and reported a range of nicotine (< 0.04–4.6 µg/m^3^), propylene glycol (< 0.04–395.0 µg/m^3^), glycerin (< 0.04–81.0 µg/m^3^), formaldehyde (21.0–55.0 µg/m^3^) and acetaldehyde (16.0–162.0 m^3^) concentrations among other constituents from use of different e-liquid formulations. Considering the differences in participant population (occasional smokers vs. smokers of greater than or equal to 10 cigarettes/day) room size (18 m^2^ vs 51.9 m^2^ and 45 m^3^ vs 136 m^3^) ventilation (0.56 vs 7.5 air exchanges/hour), and products studied, the average four-hour cumulative concentrations for prescribed or ad libitum ENDS use from the current study are remarkably consistent for propylene glycol (30.34–51.17 µg/m^3^), glycerin (70.85–110.55 µg/m^3^), and nicotine (3.45–6.14 µg/m^3^). Measured concentrations of formaldehyde (below baseline value—0.03 μg/m^3^) and acetaldehyde (below baseline value—0.52 µg/m^3^) were substantially lower than reported by one study^16^ likely due to the different products used in the two studies and our use of ventilation rates compliant with American Society of Heating, Refrigerating and Air-Conditioning Engineers (ASHRAE)/ANSI standards^31^ for the room size used. When similar ventilation rates are used^19^, results are more consistent with the current study. This study^19^ used experienced ENDS users and cigarette smokers and compared concentrations of selected air constituents in a slightly smaller room (38 m^2^ vs 51.9 m^2^ and 114 m^3^ vs 136 m^3^) using the same office ventilation rate as the current study. For prescribed and ad libitum product use^19^ the reported a range of nicotine (0.38–2.83 µg/m^3^), propylene glycol (33.06–211.51 µg/m^3^), glycerin (67.89–126.75 µg/m^2^), and formaldehyde (below baseline values) concentrations are more consistent with concentrations measured in the current study than those of^16^.

Use of conventional cigarettes resulted in greater particle number concentration than either prescribed or ad libitum use of ENDS. This was expected since conventional cigarettes generate side stream smoke between puffs, which ENDS do not. Using the prescribed puffing regimen, Group II participants exhaled a higher particle number concentration than Group I participants, which might be a function of the amount of e-liquid used. Consistent with previous conclusions of^32^, ENDS aerosols have a high proportion of volatile and semi-volatile ingredients and are temporally dynamic, making comparisons with previous studies measuring particle number concentration, tenuous at best. Combined with differences in measurement equipment, puffing regimens and products tested, our results are in general agreement with the particle number concentrations previously reported^18^ (up to 220,000 particles/cm^3^) even though a larger room with office ventilation was used, as well as^20^, (50,000 particles/cm^3^ peak and approximately 9,000 particles/cc between peaks) when a smaller room with lower ventilation rate (0.67 air exhanges/h) and four participants were used.

The statistically significant differences in measured baseline concentrations between ventilation conditions within Groups and between Groups complicated the comparisons within and between Groups. The importance of measuring amount of product used was highlighted in Group I. In Group I, during prescribed product use an average of 136.8 mg of e-liquid was used compared to ad libitum product use when an average of 205.1 mg of e-liquid was used. In both cases cumulative 4-h measured concentrations of furan and toluene were statistically significantly different from baseline values. Contrary to the amount of e-liquid consumed, 1.40 µg/m^3^ of furan was measured during prescribed product use and only 0.9 µg/m^3^ of furan was measured with ad libitum product use. A similar pattern occurred for toluene, with 0.95 µg/m^3^ measured during prescribed product use and 0.58 µg/m^3^ measured during ad libitum product use.

The results of this study should be interpreted in the context of some of its’ limitations. Comparison of constituent levels between products during ad libitum product use not only includes the inherent differences in product use, but also includes any differences between the groups. This study did not measure surface levels of any constituents so comparison of dilution and dispersion vs. absorption to surfaces was not performed. Consistent with previous studies, this study was only conducted in a single room, however different ventilation conditions were utilized, resulting in a range of aerosol mixing and distribution; it is not known how our results would be generalizable to uniquely configured rooms (non-rectangular or excessively rectangular) or other enclosed spaces (e.g., vehicles, etc.). Although multiple ventilation conditions were used, it is not practical to perform these studies for every ENDS product, therefore, we agree with previous work^19^ that data from these types of studies should be used to verify different modeling approaches. Once the modeling approaches are verified, they can be used to investigate different scenarios with minimal experimental verification. Finally, the low concentrations measured for some constituents should be interpreted with caution due to the inherent variability in: (1) their background concentrations in fresh air; and (2) variability in baseline concentrations that incorporate amounts in normal human exhaled breath^33^. Even when considering the average amount of e-liquid consumed, small concentration changes of some constituents could not be explained meaning there are other confounding factors that were not controlled in this study. This might be due to baseline measurements not incorporating the prescribed or ad libitum puffing regimen.

In summary, our findings support the importance of baseline measurements that include participants in the room, measurement of the amount of product used, and use of different ventilation levels, which were important in the interpretation of our results. Our findings indicate that under the various study conditions, use of conventional cigarettes resulted in significantly more constituents (e.g., acetaldehyde, acetone, formaldehyde, propionaldehyde, 1,3-butadiene, benzene, ethylbenzene, furan, and isoprene) and higher 4-h cumulative concentrations of measured constituents in comparison to the two ENDS tested (JL ENDS, Virginia Tobacco 5.0% nicotine by weight and VUSE Solo Original Flavor 4.8% nicotine by weight), except for propylene glycol and glycerin, which are the predominate ENDS ingredients. ENDS use resulted in as much as a 95% reduction in some measured constituents. Additionally, use of conventional cigarettes resulted in greater particle number concentrations than either prescribed or ad libitum use of either of the ENDS used in this study.

## Material and methods

The Advarra, Canada Institutional Review Board (IRB#00000971) reviewed and approved all pertinent study documents, including the experimental study protocol and informed consent document. All Study participants provided their informed consent and the study was conducted in accordance the principles and requirements of Good Clinical Practice as defined by the U.S. Food and Drug Administration^34^ and the Declaration of Helsinki. The study was listed on clinicaltrials.gov web site with the NCT number: NCT03605641.

### Study design and procedure

**Figure 4:**
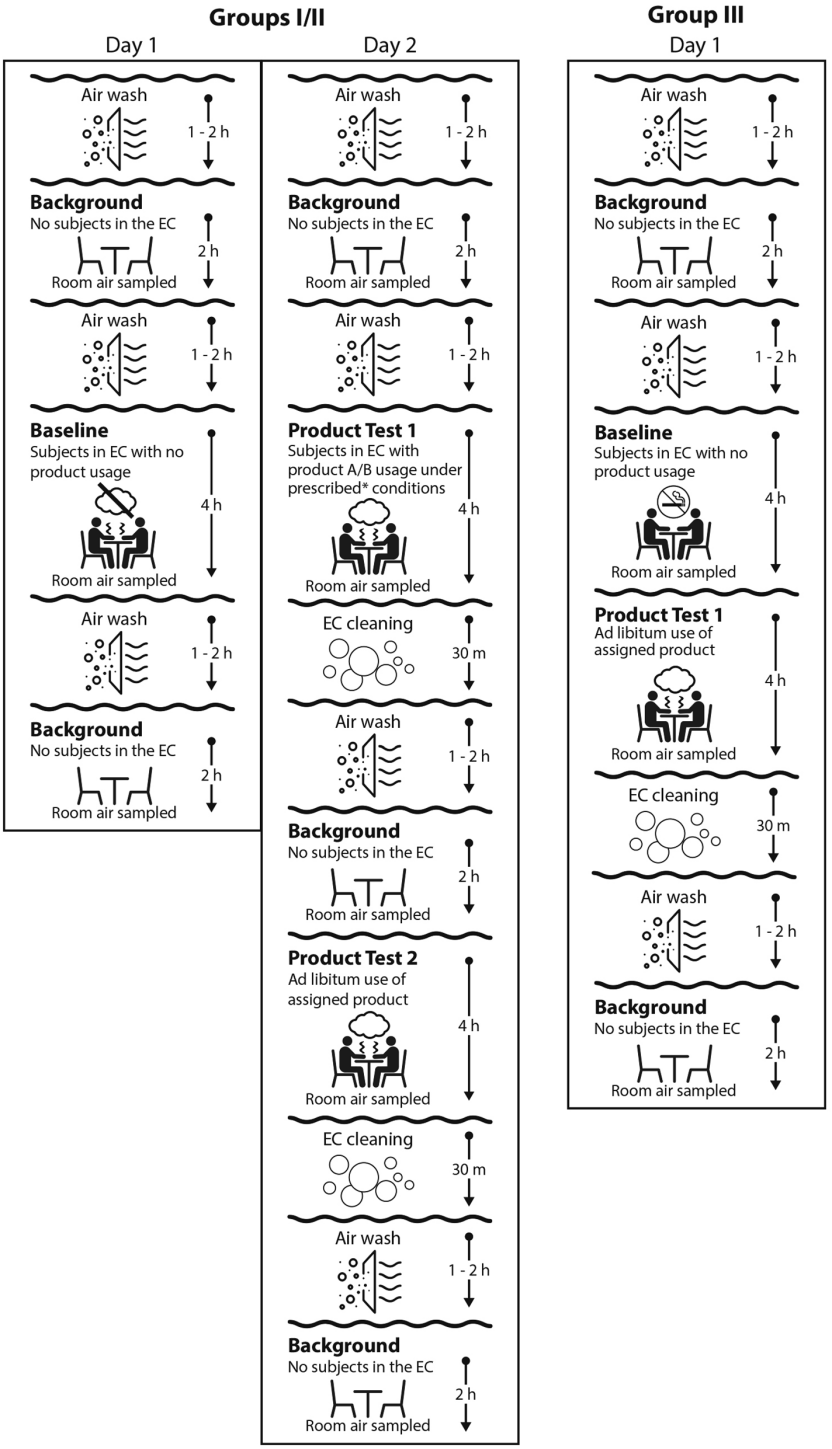
Study schedule for all groups.

This study was designed as an open-label, single center, three-arm observational study to compare concentrations of selected air constituents (chemicals) during four-hours of product use of two ENDS and conventional cigarette products using three different room ventilation conditions (residential, office and hospitality). The overall study design and scheduling is shown in Fig. 4. Following informed consent, potential participants were screened (Day − 60 to − 2) according to inclusion and exclusion criteria (Supplemental Tables S1 and S2). A total of 43 participants were enrolled in the study with 10 assigned to each of three groups (Groups I, II and III), 9 participants were unassigned and 4 failed to meet all study inclusion criteria. All enrolled participants were offered the opportunity to quit use of all tobacco products with a referral to the STOP program (Smoking Treatment for Ontario Patients). Participants in Groups I and II used a pod- or cartridge-based ENDS, respectively, under prescribed and ad libitum use conditions in each of the three ventilation conditions. Participants in Group III used their own brand of conventional cigarette only under ad libitum use conditions in each of the three ventilation conditions. Study groups were scheduled sequentially on separate days and in numerical order with the different ventilation conditions separated by at least 5 days.

All study procedures were conducted in a dedicated exposure chamber (Inflamax Research, dba Cliantha Research, Mississauga, On, Canada). The exposure chamber was validated to provide consistent environmental conditions consistent with the ASHRAE standards^31^ for residential, office, and hospitality environments. Analysis of constituents from sampling of room air was performed under 4 different conditions: (1) Background condition – sampling was performed for only carbonyls after a 2-h air wash of the exposure chamber without any study participants in the exposure chamber and was performed on all study days to account for the variable concentration of constituents in fresh air that occur throughout the day; (2) Baseline condition—sampling for all constituents was performed for four hours with participants in the exposure chamber without any product use to account for concentrations of exhaled constituents from participants^33^; (3) Prescribed product use (only Groups I and II)—sampling was performed for four hours with participants using the assigned ENDS every 30 min. Each product use consisted of 10 puffs each of 3 s. duration with an interpuff interval of 30 s.; (4) ad libitum product use (all groups)—sampling was performed for four hours with participants using their assigned product as desired. For Groups I and II product use consisted of at least 40 puffs without restrictions on puff volume or frequency. For Group III a minimum of one cigarette/hour was required.

All subjects within each group entered the exposure chamber at the same time for all baseline, prescribed and ad libitum product use. They were requested to remain seated and alert. Only drinking water was provided within the exposure chamber on an ad libitum basis. Exit and re-entry from the exposure chamber was limited to minimize airflow disruption. The post screening study schedule for each ventilation condition (second, third and fourth visit) is shown in Fig. 4. All participants returned to the clinic the evening prior to Day 1 for an overnight stay with confirmed abstinence from any tobacco or nicotine products for 10 h.

### Study participants

The study plan was to enroll approximately 30 healthy male and female conventional cigarette smokers (10 participants per group) between the ages of 21 and 65 years old. Study participants had to have a self-reported minimum daily conventional cigarette consumption rate of 10 cigarettes/day for the past 3 months, a urinary cotinine result at the Screening Visit of > 200 ng/ml and be in good general health as documented by their medical history, physical examination, assessment of vital signs and general observation.

### Products used

Participants in Group I were assigned to use a pod-based ENDS (JUUL Labs, Inc. San Francisco, CA, USA). The Virginia Tobacco flavored product (JL ENDS) contained 5.0% (by weight) United States Pharmacopeia Grade nicotine and a mixture of propylene glycol, glycerin, benzoic acid, and flavors. Participants in Group II were assigned to use a replaceable cartridge-based ENDS, VUSE Solo (Reynolds American International, Winston-Salem, NC, USA). The Original Flavor VUSE Solo e-liquid consisted of 4.8% (by weight) nicotine, propylene glycol, vegetable glycerin, flavors and water. Participants in Group III used their own store bought conventional (not hand-rolled) Canadian full-flavored cigarette product. For Groups I and II, the weight of each pod or cartridge was measured prior to and after use to measure the amount e-liquid that was used.

### Study endpoints

Room air samples were analyzed for 34 constituents (Table 2). These constituents were selected because they are typically measured for indoor air sampling, have been measured in previous studies^19,34–38^ and have validated aerosol collection and analytical methods. Additionally, 29 of the 34 constituents are listed as or are proposed for addition to the list of harmful and potentially harmful constituents in tobacco products by the U.S. Food and Drug Administration^39,40^. Primary study endpoints were changes in the concentration of the 34 constituents from baseline (no product use in exposure chamber) to product use conditions for the three study products using three different ventilation conditions (background corrected where applicable).

### Environmental chamber and air sampling

**Figure 5:**
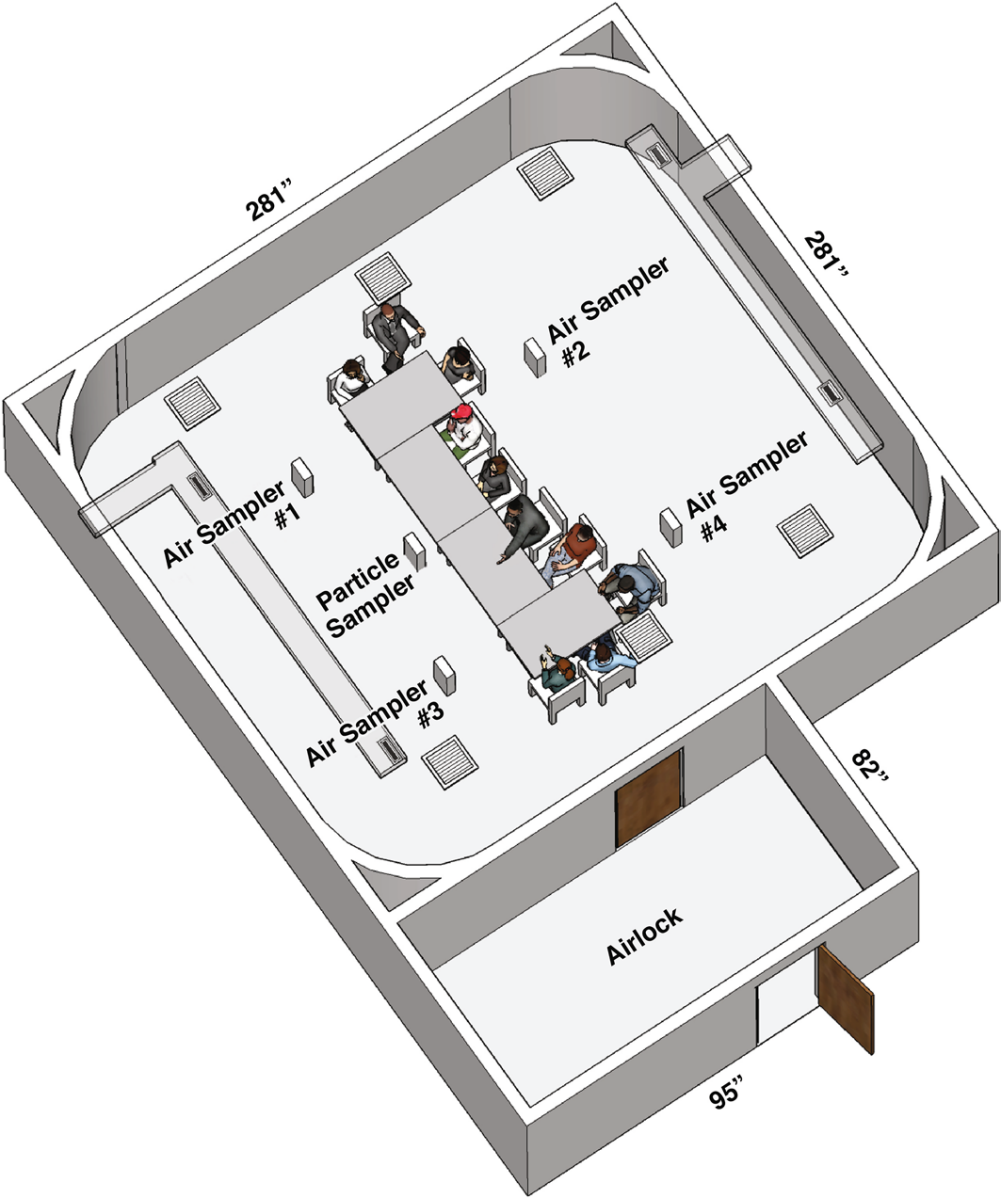
Exposure Chamber and Airlock showing air and particle sampler locations.

The environmental chamber used in this study was 51.9 m^2^ with a volume of 136 m^3^. To minimize airflow disruption in the environmental chamber due to door opening and closing, an airlock room (5.1 m^2^ and 13.4 m^3^ in volume) was directly attached to the environmental chamber and was used to control access to the environmental chamber. A schematic diagram of the airlock room and exposure chamber showing the position of study participants at tables, four room air samplers and the particle sampler is shown in Fig. 5. For each of the ventilation conditions used in this study (residential, office and hospitality), ASHRAE standards^31^ for living spaces were used (Table 1). All fresh air supplied was HEPA filtered prior to entering the exposure chamber. Temperature in the exposure chamber was maintained at 22 ± 1 °C and relative humidity (RH) was maintained at 50% ± 30% RH. Air sampler placement was specifically not designed to investigate impact of a “proximity or source effect”, but rather to provide an overall average of potential non-user exposure to exhaled aerosols in a room that was compliant with building codes^31^ at three ventilation conditions.

Based upon a previous study^19^, the exposure chamber volume and ventilation, room air was collected continuously during background (2 h), baseline (4 h) and product use conditions (4 h) for cumulative samples to ensure adequate detection levels. Measurement of particle number concentration was also performed continuously with data collected for averaging every minute. Only carbonyls were measured during background conditions. For the baseline and product use conditions, sampling and particle counting started when all study participants were seated. The sampling methods and analytical analysis have been previously published^19,41^ with details provided in the Supplemental Methods. A total of four air samplers (labelled Air Sampler #1–4) were placed 117 cm above the floor, in the general breathing zone of seated participants, to sample the room air (Fig. 5). The particle sampler was placed 81.3 cm above the floor. Particle number concentrations were obtained on a minute by minute basis from an AeroTrak model 9306 airborne particle counter (0.3 to 25 µm) (TSI, Shoreview, MN) with an isokinetic sampling inlet (Fig. 5; labeled as Particle Counter). Results are provided as particle number concentration per cm^3^.

### Statistical analysis

Descriptive statistics were provided for absolute change from baseline for each selected constituent by study group and product use condition (prescribed use and ad libitum use). Paired t-tests were used to test for the difference in constituent concentration between baseline and during product use, for all groups. All values found below the limit of detection (LOD) were analyzed at LOD. For comparison of baseline concentrations of constituents between ventilation concentrations within a group and between groups ANOVA was used. Significance for the difference in constituent concentration (baseline and during product use) was set at p < 0.05. For particle number concentration, p-values and 2-sided 90% confidence intervals (Cis) for the mean treatment difference were provided, in addition to descriptive statistics. All analyses were performed with SAS 9.4 (SAS Institute, Cary, NC, USA).

## Supplementary Information

Supplementary Information.
