# Supplementary material for: Room air constituent concentrations from use of electronic nicotine delivery systems and cigarettes using different ventilation conditions
Source: Sci Rep. 2021 Jan 18;11:1736. doi: 10.1038/s41598-021-80963-9 (PMC7814121; doi:10.1038/s41598-021-80963-9)
Supplement: Supplementary file 1 — Supplementary Information. [file 41598_2021_80963_MOESM1_ESM.pdf]

Room Air Constituent Concentrations from Use of Electronic Nicotine Delivery Systems and Cigarettes using Different Ventilation Conditions

Michael J. Oldham, Anil Sehgal, Gal Cohen, Joey Chen, Blair Evans, Daniel Heraldez

Supplemental Information

Contents

Supplementary Methods 6

Table S1. Participant Inclusion Criteria: Subjects satisfied the following criteria before being tentatively enrolled into the study.......... 8

Table S2. Participant Exclusion Criteria: Subjects who met any of the following exclusion criteria were not tentatively enrolled in the study. 9

Table S3. Chemicals and validated analytical methods used for their detection in room air samples. 10

Table S4. Mean (±SD) cumulative (four-hour) baseline constituent concentrations (N=4) for each group and ventilation for any constituent detected above LOQ for at least one puffing regimen for any group. Statistically significant differences ($p < 0.05$) from one-way ANOVA found in baseline concentrations (N=4) between ventilation conditions are denoted with an asterisk. 11

Figure S1. Mean background concentrations of 2,5 Dimethylbenzaldehyde measured prior to and after Group I (solid circles), Group II (solid squares), and Group III (solid triangles) product use at the residential ventilation condition. 12

Figure S2. Mean background concentrations of 2-Butanone measured prior to and after Group I (solid circles), Group II (solid squares), and Group III (solid triangles) product use at the residential ventilation condition. 12

Figure S3. Mean background concentrations of Acetaldehyde measured prior to and after Group I (solid circles), Group II (solid squares), and Group III (solid triangles) product use at the residential ventilation condition. 13

Figure S4. Mean background concentrations of Acetone measured prior to and after Group I (solid circles), Group II (solid squares), and Group III (solid triangles) product use at the residential ventilation condition. Error bars represent the standard deviation and maybe obscured by the data point. 13

Figure S5. Mean background concentrations of Acrolein measured prior to and after Group I (solid circles), Group II (solid squares), and Group III (solid triangles) product use at the residential ventilation condition. 14

Figure S6. Mean background concentrations of Benzaldehyde measured prior to and after Group I (solid circles), Group II (solid squares), and Group III (solid triangles) product use at the residential ventilation condition. 14

Figure S7. Mean background concentrations of Butyraldehyde measured prior to and after Group I (solid circles), Group II (solid squares), and Group III (solid triangles) product use at the residential ventilation condition. 15

Figure S8. Mean background concentrations of Crotonaldehyde measured prior to and after Group I (solid circles), Group II (solid squares), and Group III (solid triangles) product use at the residential ventilation condition. 15

Figure S9. Mean background concentrations of Formaldehyde measured prior to and after Group I (solid circles), Group II (solid squares), and Group III (solid triangles) product use at the residential ventilation condition. 16

Figure S10. Mean background concentrations of Hexaldehyde measured prior to and after Group I (solid circles), Group II (solid squares), and Group III (solid triangles) product use at the residential ventilation condition. 16

Figure S11. Mean background concentrations of Isovaleraldehyde measured prior to and after Group I (solid circles), Group II (solid squares), and Group III (solid triangles) product use at the residential ventilation condition. 17

Figure S12. Mean background concentrations of Propionaldehyde measured prior to and after Group I (solid circles), Group II (solid squares), and Group III (solid triangles) product use at the residential ventilation condition. 17

Figure S13. Mean background concentrations of Valeraldehyde measured prior to and after Group I (solid circles), Group II (solid squares), and Group III (solid triangles) product use at the residential ventilation condition. 18

Figure S14. Mean background concentrations of m&p-Tolualdehyde measured prior to and after Group I (solid circles), Group II (solid squares), and Group III (solid triangles) product use at the residential ventilation condition. 18

Figure S15. Mean background concentrations of Tolualdehyde measured prior to and after Group I (solid circles), Group II (solid squares), and Group III (solid triangles) product use at the residential ventilation condition. 19

Figure S16. Mean background concentrations of 2,5 Dimethylbenzaldehyde measured prior to and after Group I (solid circles), Group II (solid squares), and Group III (solid triangles) product use at the office ventilation condition. 19

Figure S17. Mean background concentrations of 2-Butanone measured prior to and after Group I (solid circles), Group II (solid squares), and Group III (solid triangles) product use at the office ventilation condition. 20

Figure S18. Mean background concentrations of Acetaldehyde measured prior to and after Group I (solid circles), Group II (solid squares), and Group III (solid triangles) product use at the office ventilation condition. 20

Figure S19. Mean background concentrations of Acetone measured prior to and after Group I (solid circles), Group II (solid squares), and Group III (solid triangles) product use at the office ventilation condition. 21

Figure S20. Mean background concentrations of Acrolein measured prior to and after Group I (solid circles), Group II (solid squares), and Group III (solid triangles) product use at the office ventilation condition. 21

Figure S21. Mean background concentrations of Benzaldehyde measured prior to and after Group I (solid circles), Group II (solid squares), and Group III (solid triangles) product use at the office ventilation condition. 22

Figure S22. Mean background concentrations of Butyraldehyde measured prior to and after Group I (solid circles), Group II (solid squares), and Group III (solid triangles) product use at the office ventilation condition. 22

Figure S23. Mean background concentrations of Crotonaldehyde measured prior to and after Group I (solid circles), Group II (solid squares), and Group III (solid triangles) product use at the office ventilation condition. 23

Figure S24. Mean background concentrations of Formaldehyde measured prior to and after Group I (solid circles), Group II (solid squares), and Group III (solid triangles) product use at the office ventilation condition. 23

Figure S25. Mean background concentrations of Hexaldehyde measured prior to and after Group I (solid circles), Group II (solid squares), and Group III (solid triangles) product use at the office ventilation condition. 24

Figure S26. Mean background concentrations of Isovaleraldehyde measured prior to and after Group I (solid circles), Group II (solid squares), and Group III (solid triangles) product use at the office ventilation condition. 24

Figure S27. Mean background concentrations of Propionaldehyde measured prior to and after Group I (solid circles), Group II (solid squares), and Group III (solid triangles) product use at the office ventilation condition. 25

Figure S28. Mean background concentrations of Valeraldehyde measured prior to and after Group I (solid circles), Group II (solid squares), and Group III (solid triangles) product use at the office ventilation condition. 25

Figure S29. Mean background concentrations of m&p-Tolualdehyde measured prior to and after Group I (solid circles), Group II (solid squares), and Group III (solid triangles) product use at the office ventilation condition. 26

Figure S30. Mean background concentrations of Tolualdehyde measured prior to and after Group I (solid circles), Group II (solid squares), and Group III (solid triangles) product use at the office ventilation condition. 26

Figure S31. Mean background concentrations of 2,5 Dimethylbenzaldehyde measured prior to and after Group I (solid circles), Group II (solid squares), and Group III (solid triangles) product use at the hospitality ventilation condition. 27

Figure S32. Mean background concentrations of 2-Butanone measured prior to and after Group I (solid circles), Group II (solid squares), and Group III (solid triangles) product use at the hospitality ventilation condition. 27

Figure S33. Mean background concentrations of Acetaldehyde measured prior to and after Group I (solid circles), Group II (solid squares), and Group III (solid triangles) product use at the hospitality ventilation condition. 28

Figure S34. Mean background concentrations of Acetone measured prior to and after Group I (solid circles), Group II (solid squares), and Group III (solid triangles) product use at the hospitality ventilation condition. 28

Figure S35. Mean background concentrations of Acrolein measured prior to and after Group I (solid circles), Group II (solid squares), and Group III (solid triangles) product use at the hospitality ventilation condition. 29

Figure S36. Mean background concentrations of Benzaldehyde measured prior to and after Group I (solid circles), Group II (solid squares), and Group III (solid triangles) product use at the hospitality ventilation condition. 29

Figure S37. Mean background concentrations of Butyraldehyde measured prior to and after Group I (solid circles), Group II (solid squares), and Group III (solid triangles) product use at the hospitality ventilation condition. 30

Figure S38. Mean background concentrations of Crotonaldehyde measured prior to and after Group I (solid circles), Group II (solid squares), and Group III (solid triangles) product use at the hospitality ventilation condition. 30

Figure S39. Mean background concentrations of Formaldehyde measured prior to and after Group I (solid circles), Group II (solid squares), and Group III (solid triangles) product use at the hospitality ventilation condition. 31

Figure S40. Mean background concentrations of Hexaldehyde measured prior to and after Group I (solid circles), Group II (solid squares), and Group III (solid triangles) product use at the hospitality ventilation condition. 31

Figure S41. Mean background concentrations of Isovaleraldehyde measured prior to and after Group I (solid circles), Group II (solid squares), and Group III (solid triangles) product use at the hospitality ventilation condition. 32

Figure S42. Mean background concentrations of Propionaldehyde measured prior to and after Group I (solid circles), Group II (solid squares), and Group III (solid triangles) product use at the hospitality ventilation condition. 32

Figure S43. Mean background concentrations of Valeraldehyde measured prior to and after Group I (solid circles), Group II (solid squares), and Group III (solid triangles) product use at the hospitality ventilation condition. 33

Figure S44. Mean background concentrations of m&p-Tolualdehyde measured prior to and after Group I (solid circles), Group II (solid squares), and Group III (solid triangles) product use at the hospitality ventilation condition. 33

Figure S45. Mean background concentrations of Tolualdehyde measured prior to and after Group I (solid circles), Group II (solid squares), and Group III (solid triangles) product use at the hospitality ventilation condition. 34

Supplementary Methods

Air Sampling

Each of the four air samplers was a custom seven-port parallel air sampler (Enthalpy Analytical, Inc., Durham, NC, USA) designed to continuously sample a defined amount of air through each port using a vacuum pump. Each of the seven-ports was used for a specific constituent analysis.

Nicotine, propylene glycol and glycerin were collected on XAD-7 (SKC 226-95) adsorbent tubes with a target flow rate of 900 mL/min on two independent ports. Fifteen selected carbonyls (2, 5-dimethylbenzaldehyde, acetaldehyde, acetone, acrolein, benzaldehyde, butyraldehyde, crotonaldehyde, formaldehyde, hexanaldehyde, isovaleraldehyde, m&p-tolualdehyde, methyl ethyl ketone, o-tolualdehyde, propionaldehyde and valeraldehyde) were collected on 2,4-dinitrophenyhydrazine (DNPH)-coated silica gel adsorbent tubes (SKC 226-119) with a target flow rate of 200 mL/min and were sampled on two independent ports. Twelve selected VOCs (1,3-butadiene, 2-nitropropane, benzene, ethylbenzene, ethylene oxide, furan, isoprene, nitromethane, propylene oxide, toluene, vinyl acetate, and vinyl chloride) were collected on SVI thermal desorption tubes (Perkin-Elmer Inc. Waltham, MA, USA) with a target flow rate of 60 mL/min from one port. Four selected trace metals (arsenic, cadmium, chromium, and nickel) were collected on quartz filters (SKC 225-401) with a target flow rate of 1700 mL/min on two independent ports. Airflow through the ports used to sample nicotine, propylene glycol, glycerin, selected carbonyls and selected VOCS was controlled with a critical orifice. A fine needle value was used to control airflow through each of the two ports used for sampling selected metals. Flows were measured prior to and after sample collection with the mean used to calculate air constituent concentrations. Each of the four air samplers was tested for leaks by plugging all sampling media inlets and pulling a vacuum greater than 38 cm of Hg. A decay of ≤ 1.25 cm of Hg over 30 sec. was considered acceptable.

Analytical analysis

All analysis was conducted by Enthalpy Analytical Inc. (Durham, NC, USA) an ISO 17025 accredited laboratory. Nicotine collected on the XAD-7 adsorbant tubes was analyzed following the ISO 16200-1 method[1] using an Agilent Model 7890 Gas Chromatograph (GC) integrated

with an Agilent Model 5975C Mass Selective Detector (Agilent Technologies, Santa Clara, CA, USA). Propylene Glycol and glycerin collected on the XAD-7 adsorbant tubes were also analyzed following ISO 16200-1 method[1] but used an Agilent Model 6890 GC with a Flame Ionization Detector. The selected carbonyls collected on DNPH-coated silica gel adsorbant tubes were analyzed following the ISO 16000-3 method[2] using an Agilent Model 1100 High Performance Liquid Chromatograph with a DAD-UV detector which was operated at 365nm (ISO 2011a). Selected VOCs collected on SVI thermal desorption tubes were analyzed following the ISO 16000-6 method[3] using an Agilent Model 6890 GC coupled to an Agilent Model 5973 Mass Selective Detector. The quartz filters were analyzed for the four trace metals using Inductively Coupled Plasma-Mass Spectrometry with a Perkin Elmer DRC-e ICP-MS in accordance with the EPA IO2.1 and 3.5 method[4, 5].

References

1. ISO: International Organization for Standardization. 2001. 16200-1. Workplace Air Quality—Sampling and Analysis of Volatile Organic Compounds by Solvent Desorption/Gas Chromatography—Part 1: Pumped Sampling Method; ISO: Geneva, Switzerland.
2. ISO: International Organization for Standardization. 2011a. 16000-3. Indoor Air—Part 3: Determination of Formaldehyde and Other Carbonyl Compounds in Indoor Air and Test Chamber Air—Active Sampling Method; ISO: Geneva, Switzerland.
3. ISO: International Organization for Standardization. 2011b. 16000-6. Indoor Air—Part 6: Determination of volatile Organic Compounds in Indoor and Test Chamber Air by Active Sampling on Tenax TA Sorbent, Thermal Desorption and Gas Chromatography Using MS or MS-FID; ISO: Geneva, Switzerland.
4. EPA: US Environmental Protection Agency. 1999a. Compendium Method IO-2.1. Sampling of Ambient Air for Total Suspended Particulate Matter (Spm) and PM10 Using High Volume (Hv) Sampler; EPA: Washington, DC, USA.
5. EPA: US Environmental Protection Agency. 1999b. Compendium Method IO-3.5. Determination of Metals in Ambient Particulate Matter Using Inductively Coupled Plasma/ Mass Spectrometry (ICP/MS); EPA: Washington, DC, USA.

Table S1. Participant Inclusion Criteria: Subjects satisfied the following criteria before being tentatively enrolled into the study

Item	Criteria
1	Informed of the nature of the study and had agreed to and were able to read, review, and sign the informed consent document (in English) prior to the first study procedure. Therefore, the volunteer must have had the ability to read and communicate in English in order to participate in the study
2	Subjects screened as a part of an IRB-approved General Screening Protocol at the CRO site may have been included in this study without additional Screening procedures provided all the required screening procedures were completed within 60 days prior to Clinic Visit 1.
3	Healthy male or female volunteer aged 21 -65 years at the time of Clinic Visit 1
4	Demonstrated a positive urine cotinine result at Screening(Screening Visit) of >200 ng/mL.
5	Judged by the Investigator to be in good general health as documented by the medical history, physical examination (including but may not have been limited to an evaluation of the cardiovascular, gastrointestinal, respiratory and central nervous systems), vital sign assessments, electrocardiogram (ECG), clinical laboratory assessments, and by general observations. Any abnormalities or deviations outside the normal ranges for any clinical testing (laboratory tests, ECG, vital signs) could have been repeated at the discretion of the Investigator and judged to be not clinically significant for study participation.
6	Agreed to abide by the study restrictions and return for the required assessments.
7	Had a self-reported daily conventional(combustible)cigarette consumption rate of a minimum of10 cigarettes per day for a minimum of 3 months prior to Screening Visit.

Table S2. Participant Exclusion Criteria: Subjects who met any of the following exclusion criteria were not tentatively enrolled in the study.

Item	Criteria
1	Reported receiving any investigational product within 30days prior to Screening (Screening Visit).
2	Reported any presence or history of a clinically significant disorder involving the cardiovascular, respiratory, renal, urologic, gastrointestinal, hepatic, immunologic, hematologic, endocrine, oncologic or neurologic system(s) or psychiatric disease as determined by the Investigator.
3	Presence of any clinically significant results from laboratory tests, physical examinations, vital signs assessments, and electrocardiograms, as judged by the Investigator.
4	Reported a clinically significant illness during the 30 days prior to enrollment, as determined by the Investigator.
5	Reported a history of drug or alcohol addiction or abuse within the past 1 year.
6	Demonstrated a positive screen for alcohol or drugs of abuse at Screening (Screening Visit) or check-in at Clinical Visit 1, 2 or 3.
7	Demonstrated a positive test for human immunodeficiency virus (HIV), hepatitis B surface antigen (HBsAg), or hepatitis C virus (anti-HCV).
8	Body mass index (BMI) greater than 40 kg/m2or less than 18 kg/m2at Screening (Screening Visit).
9	Reported use of prescription anti-diabetic medication and/or insulin therapy within 12months of Screening (Screening Visit).
10	Reported taking medication for depression or asthma within 6 months of Screening (Screening Visit).
11	Reported use of prescription or over-the-counter bronchodilator medication (e.g., inhaled or oral β-agonists) within 6 months of Screening (Screening Visit).
12	Breast-feeding or pregnant female subjects (confirmed by a positive pregnancy test). Female subjects, who were considered women of child bearing potential (WOCBP) and sexually active, were willing and able to use an acceptable method of contraception from Screening (Screening Visit) through the end of the study.
13	Allergic to propylene glycol or vegetable glycerin.
14	Were or had a first-degree relative (i.e., parent, sibling or child) who was a current employee of the CRO, Sponsor, or Site.

Table S3. Chemicals and validated analytical methods used for their detection in room air samples.

Chemicals	Validated Analytical Method
1,3 butadiene	ISO 16000-6
2,5-dimethylbenzaldehyde	ISO 160003 & EPA TO-11A
2-nitropropane	ISO 16000-6
Acetaldehyde	ISO 160003 & EPA TO-11A
Acetone	ISO 160003 & EPA TO-11A
Acrolein	ISO 160003 & EPA TO-11A
Arsenic[1]	EPA IO-2.1 & 3.5
Benzaldehyde	ISO 160003 & EPA TO-11A
Benzene	ISO 16000-6
Butyraldehyde	ISO 160003 & EPA TO-11A
Cadmium[1]	EPA IO-2.1 & 3.5
Carbon monoxide	ISO 160003 & EPA TO-11A
Chromium[1]	EPA IO-2.1 & 3.5
Crotonaldehyde	ISO 160003 & EPA TO-11A
Ethylbenzene	ISO 16000-6
Ethylene oxide	ISO 16000-6
Formaldehyde	ISO 160003 & EPA TO-11A
Furan	ISO 16000-6
Glycerin	ISO 16200
Hexanaldehyde	ISO 160003 & EPA TO-11A
Isoprene	ISO 16000-6
Isovaleraldehyde	ISO 160003 & EPA TO-11A
m&p tolualdehyde	ISO 160003 & EPA TO-11A
Methyl ethyl ketone (MEK)	ISO 160003 & EPA TO-11A
Nickel[1]	EPA IO-2.1 & 3.5
Nicotine	ISO 16200
Nitromethane	ISO 16000-6
o-tolualdehyde	ISO 160003 & EPA TO-11A
Propionaldehdye	ISO 160003 & EPA TO-11A
Propylene glycol	ISO 16200
Propylene oxide	ISO 16000-6
Toluene	ISO 16000-6
Valeraldehyde	ISO 160003 & EPA TO-11A
Vinyl acetate	ISO 16000-6
Vinyl chloride[1]	ISO 16000-6

1. Not detected above limit of quantification for any group at any ventilation.

Table S4. Mean (±SD) cumulative (four-hour) baseline constituent concentrations (N=4) for each group and ventilation for any constituent detected above LOQ for at least one puffing regimen for any group. Statistically significant differences ($p < 0.05$) from one-way ANOVA found in baseline concentrations (N=4) between ventilation conditions are denoted with an asterisk.

Constituent	LOQ (μg/m^3)	Baseline Measurements (μg/m^3)								
		Group I			Group II			Group III		
		Residential	Office	Hospitality	Residential	Office	Hospitality	Residential	Office	Hospitality
Nicotine*	1.87	0.59 ± 0.38	1.03 ± 0.31	0.38 ± 0.01	5.79 ± 3.98	0.39 ±0.02	1.78 ± 2.35	0.60 ± 0.02	0.38 ± 0.01	1.74 ± 0.73
Propylene Glycol	31.93	10.91 ± 5.72	23.08 ± 20.94	16.97 ± 10.35	12.54 ± 3.36	10.50 ± 2.59	26.83 ± 26.45	37.60 ± 9.92	21.38 ± 9.96	24.33 ± 9.99
Glycerin	33.66	26.40 ± 15.34	18.93 ± 9.55	12.75 ± 0.81	35.20 ± 1.04	18.33 ± 0.85	51.8 ± 63.09	14.78 ± 1.27	16.93 ± 1.14	46.50 ± 32.65
Acetaldehyde	24.82	6.33 ± 2.42	7.61 ± 0.18	8.08 ± 0.38	7.22 ± 0.64	7.73 ± 0.35	7.46 ± 0.35	8.35 ± 0.7	9.62 0.84	6.52 ± 0.41
Acetone*	24.71	35.15 ± 1.93	30.35 ± 3.3	27.13 ± 0.3	38.30 ± 2.5	26.75 ± 1.87	26.55 ± 2.05	27.40 ± 1.30	33.38 ± 3.56	29.85 ± 2.63
Acrolein	24.82	8.86 ± 0.57	8.76 ± 0.32	8.62 ± 0.19	8.58 ± 0.24	8.61 ± 0.26	8.70 ± 0.33	8.71 ± 0.26	8.61 ± 0.16	8.67 ± 0.23
Benzaldehyde	28.82	10.42 ± 0.66	10.28 ± 0.37	10.13 ± 0.23	10.07 ± 0.27	10.13 ± 0.33	10.22 ± 0.4	10.25 ± 0.31	10.11 ± 0.2	10.19 ± 0.29
Butyraldehyde	25.03	17.69 ± 1.13	17.50 ± 0.65	17.22 ± 0.39	17.14 ± 0.46	17.20 ± 0.54	17.38 ± 0.64	17.38 ± 0.5	17.19 ± 0.32	17.32 ± 0.48
Crotonaldehyde	25.03	4.99 ± 0.32	4.94 ± 0.18	4.86 ± 0.11	4.84 ± 0.13	4.85 ± 0.15	4.90 ± 0.18	7.82 ± 0.23	4.85 ± 0.09	4.89 ± 0.13
Formaldehyde	24.71	10.63 ± 0.39	9.29 ± 1.06	9.03 ± 0.81	9.78 ± 0.24	9.83 ± 0.49	9.46 ±0.62	10.07 ± 0.63	11.55 ± 0.34	8.31 ± 0.5
Hexanaldehyde	24.82	12.90 ± 0.82	12.74 ± 0.45	12.54 ± 0.28	12.47 ± 0.34	12.53 ± 0.37	12.64 ± 0.47	12.68 ± 0.36	12.51 ± 0.23	12.63 ± 0.34
Isovaleraldehyde	24.82	6.95 ± 0.44	6.86 ± 0.25	6.76 ± 0.15	6.73 ± 0.18	6.75 ± 0.21	6.82 ± 0.25	6.82 ± 0.2	6.75 ± 0.12	6.80 ± 0.18
Methyl Ethyl Ketone	24.189	6.09 ± 0.78	5.72 ± 0.31	6.22 ± 1.0	5.67 ± 0.12	5.78 ± 0.42	6.08 ± 0.66	5.58 ± 0.16	6.25 ± 0.54	6.65 ± 1.20
m&p tolualdehyde	49.64	7.32 ± 0.47	7.24 ± 0.26	7.12 ± 0.16	7.09 ± 0.19	7.12 ± 0.22	7.19 ± 0.27	7.20 ± 0.21	7.11 ± 0.13	7.16 ± 0.19
o-tolualdehyde	25.03	7.96 ± 0.51	7.87 ± 0.29	7.75 ± 0.17	7.71 ± 0.21	7.74 ± 0.24	7.81 ± 0.29	7.82 ± 0.23	7.73 ± 0.14	7.79 ± 0.21
Propionaldehyde	24.82	7.79 ± 1.77	6.90 ± 0.45	6.31 ± 0.35	6.34 ± 0.1	6.23 ± 0.31	8.44 ± 2.41	7.67 ± 0.43	9.84 ± 2.01	11.04 ± 1.34
Valeraldehyde	24.82	6.66 ± 0.35	6.36 ± 0.23	6.26 ± 0.14	6.23 ± 0.17	6.26 ± 0.19	6.32 ± 0.24	6.32 ± 0.18	6.26 ± 0.12	6.30 ± 0.17
2,5-dimethylbenzaldehyde	25.03	22.09 ± 1.43	21.83 ± 0.78	21.48 ± 0.46	21.38 ± 0.58	21.46 ± 0.66	21.67 ± 0.8	21.70 ± 0.62	21.44 ± 0.39	21.61 ± 0.57
1,3-Butadiene	0.3	0.45 ± 0.06	0.48 ± 0.05	0.35 ± 0.06	0.35 ± 0.06	0.38 ± 0.05	0.38 ± 0.05	0.73 ± 0.05	0.4 ±0.0	0.38 0.05
2-nitropropane	0.7	0.7 ± 0.0	0.7 ± 0.0	0.7 ± 0.0	0.7 ± 0.0	0.7 ± 0.0	0.7 ± 0.0	0.73 ± 0.05	0.7 ± 0.0	0.7 ± 0.0
Benzene	0.3	0.33 0.05	0.4 ± 0.0	0.53 ± 0.13	0.35 ± 0.06	0.38 ± 0.05	0.43 ± 0.13	0.78 ± 0.05	1.33 ± 0.13	0.43 ± 0.1
Ethylbenzene*	0.3	0.7 ± 0.0	0.73 ± 0.05	0.50 ± 0.08	1.48 0.17	0.4 ± 0.0	0.75 ± 0.1	0.43 ± 0.05	1.18 ± 0.1	0.38 ± 0.1
Ethylene Oxide	3.4	3.48 ± 0.1	3.55 ± 0.06	3.50 ± 0.08	3.53 0.05	3.50 ± 0.08	3.53 ± 0.1	3.73 ± 0.26	3.53 ± 0.05	3.55 ± 0.13
Furan	0.7	0.7 ± 0.0	0.7 ± 0.0	0.7 ± 0.0	0.7 ± 0.0	0.7 ± 0.0	0.7 ± 0.0	2.33 ± 1.09	0.7 ± 0.0	0.7 ± 0.0
Isoprene*	0.3	2.90 ± 0.61	2.65 ± 0.5	1.65 ± 0.41	3.10 ± 0.67	1.73 ± 0.41	2.08 ± 0.33	4.63 ± 0.54	2.13 ± 0.99	1.93 ± 0.19
Nitromethane	0.7	0.7 ± 0.0	0.7 ± 0.0	0.7 ± 0.0	0.85 ± 0.1	0.7 ± 0.0	0.7 ± 0.0	0.73 ± 0.05	0.7 ± 0.0	0.7 ± 0.0
Propylene Oxide	3.4	3.48 ± 0.1	3.55 ± 0.06	3.50 ± 0.08	3.53 ± 0.05	3.50 ± 0.08	3.53 ± 0.1	3.73 ± 0.26	3.53 ± 0.05	3.55 ± 0.13
Toluene*	0.3	1.00 ± 0.08	1.75 ± 0.13	1.90 ± 0.08	2.5 ± 0.2	1.05 ± 0.06	2.10 ± 0.08	1.25 ± 0.3	4.2 ± 0.2	0.85 ± 0.13
Vinyl acetate	0.7	0.7 ± 0.0	0.7 ± 0.0	0.7 ± 0.0	0.7 ± 0.0	0.7 ± 0.0	0.7 ± 0.0	0.73 ± 0.05	0.7 ± 0.0	0.7 ± 0.0

LOQ: Limit of Quantification is based upon the amount of air sampled and is a composite average from all 4 air samplers used in the six runs.

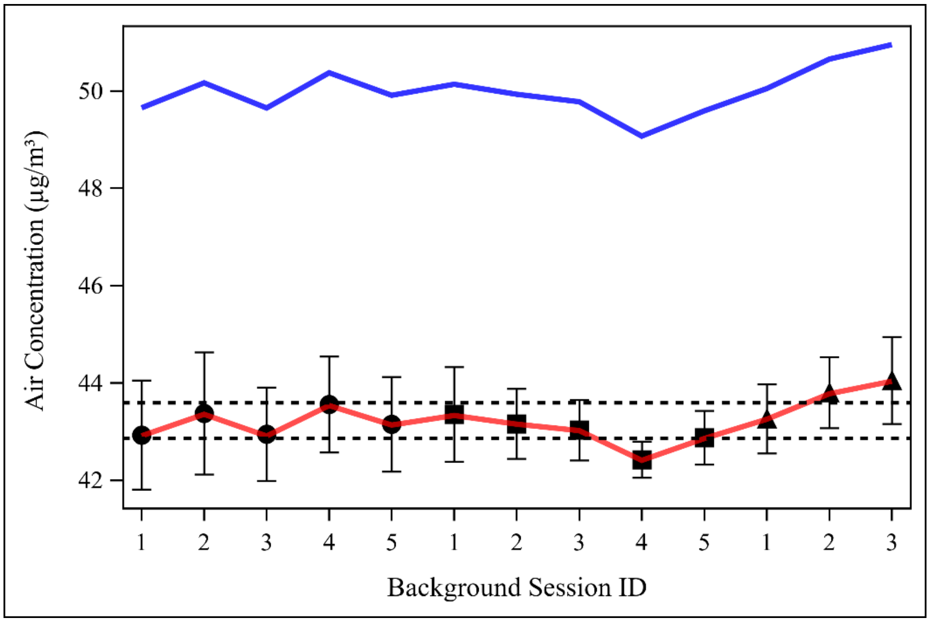

Figure S1. Mean (N=4) background concentrations of 2,5 Dimethylbenzaldehyde measured prior to and after Group I (solid circles), Group II (solid squares), and Group III (solid triangles) product use at the residential ventilation condition. Error bars represent the standard deviation and maybe obscured by the data point. The 90% Confidence intervals are shown by the dashed lines with the Limit of Detection shown in red and the Limit of Quantification shown in blue.

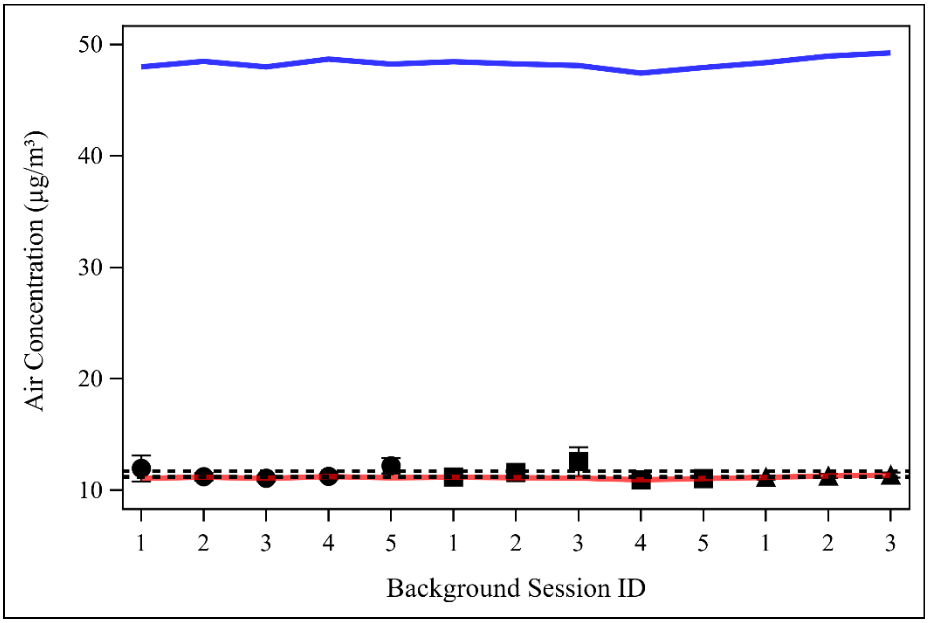

Figure S2. Mean (N=4) background concentrations of 2-Butanone measured prior to and after Group I (solid circles), Group II (solid squares), and Group III (solid triangles) product use at the residential ventilation condition. Error bars represent the standard deviation and maybe obscured by the data point. The 90% Confidence intervals are shown by the dashed lines with the Limit of Detection shown in red and the Limit of Quantification shown in blue.

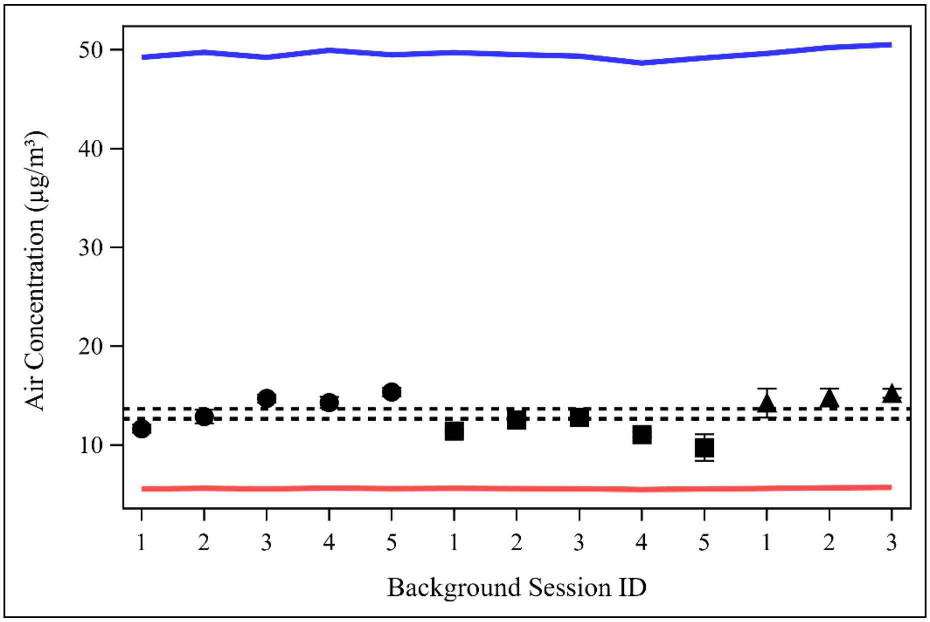

Figure S3. Mean (N=4) background concentrations of Acetaldehyde measured prior to and after Group I (solid circles), Group II (solid squares), and Group III (solid triangles) product use at the residential ventilation condition. Error bars represent the standard deviation and maybe obscured by the data point. The 90% Confidence intervals are shown by the dashed lines with the Limit of Detection shown in red and the Limit of Quantification shown in blue.

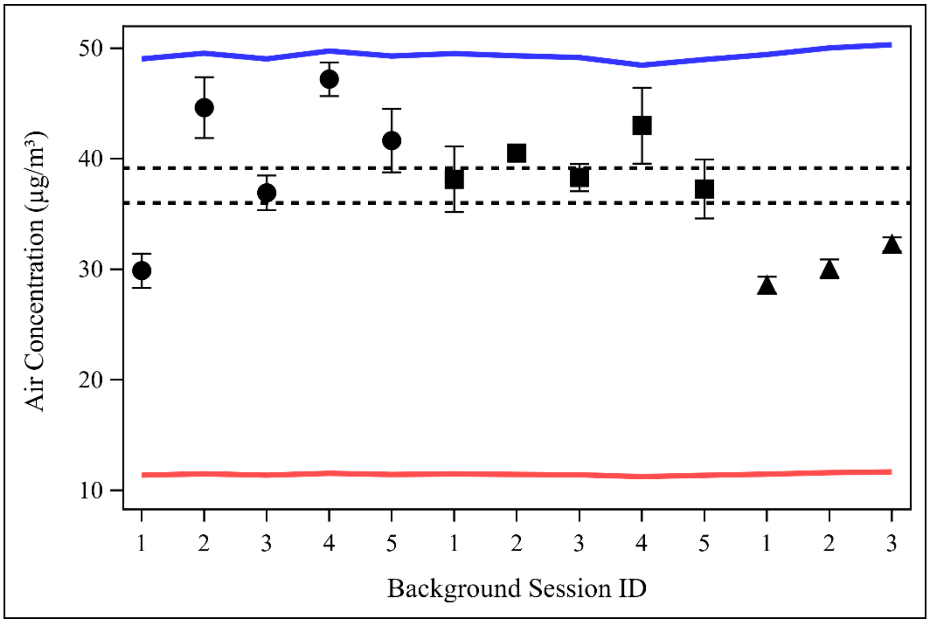

Figure S4. Mean (N=4) background concentrations of Acetone measured prior to and after Group I (solid circles), Group II (solid squares), and Group III (solid triangles) product use at the residential ventilation condition. Error bars represent the standard deviation and maybe obscured by the data point. The 90% Confidence intervals are shown by the dashed lines with the Limit of Detection shown in red and the Limit of Quantification shown in blue.

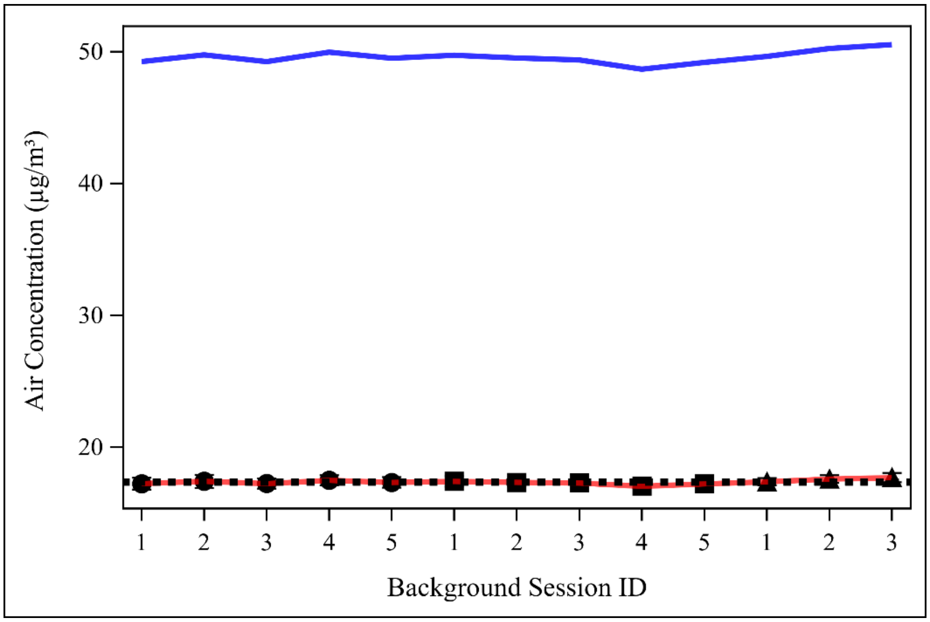

Figure S5. Mean (N=4) background concentrations of Acrolein measured prior to and after Group I (solid circles), Group II (solid squares), and Group III (solid triangles) product use at the residential ventilation condition. Error bars represent the standard deviation and maybe obscured by the data point. The 90% Confidence intervals are shown by the dashed lines with the Limit of Detection shown in red and the Limit of Quantification shown in blue.

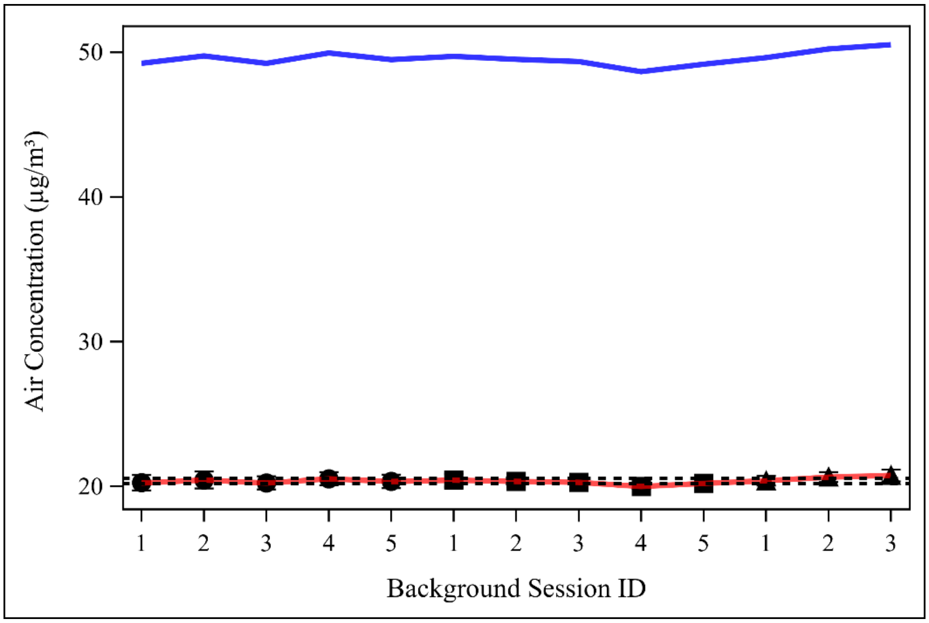

Figure S6. Mean (N=4) background concentrations of Benzaldehyde measured prior to and after Group I (solid circles), Group II (solid squares), and Group III (solid triangles) product use at the residential ventilation condition. Error bars represent the standard deviation and maybe obscured by the data point. The 90% Confidence intervals are shown by the dashed lines with the Limit of Detection shown in red and the Limit of Quantification shown in blue.

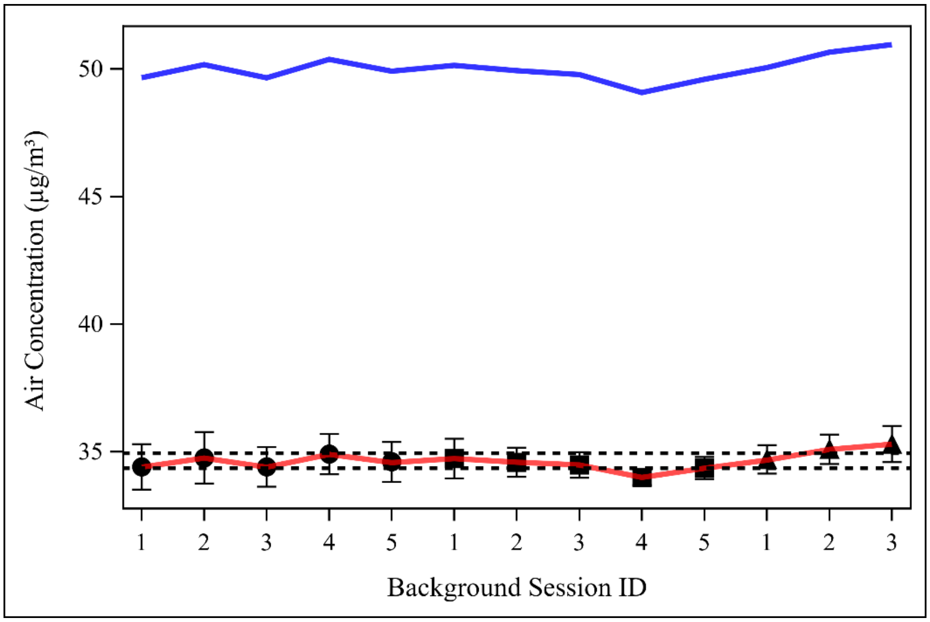

Figure S7. Mean (N=4) background concentrations of Butyraldehyde measured prior to and after Group I (solid circles), Group II (solid squares), and Group III (solid triangles) product use at the residential ventilation condition. Error bars represent the standard deviation and maybe obscured by the data point. The 90% Confidence intervals are shown by the dashed lines with the Limit of Detection shown in red and the Limit of Quantification shown in blue.

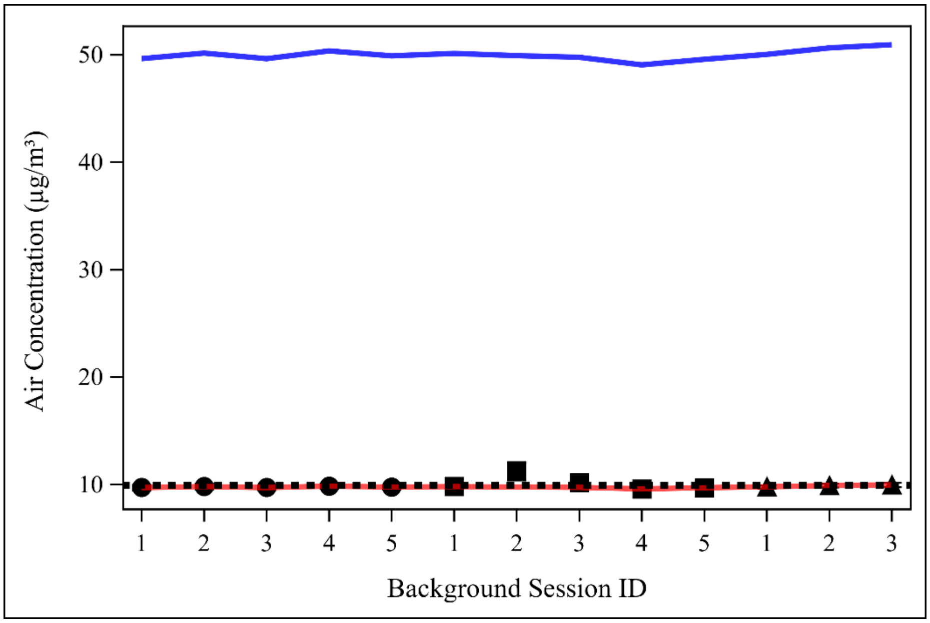

Figure S8. Mean (N=4) background concentrations of Crotonaldehyde measured prior to and after Group I (solid circles), Group II (solid squares), and Group III (solid triangles) product use at the residential ventilation condition. Error bars represent the standard deviation and maybe obscured by the data point. The 90% Confidence intervals are shown by the dashed lines with the Limit of Detection shown in red and the Limit of Quantification shown in blue.

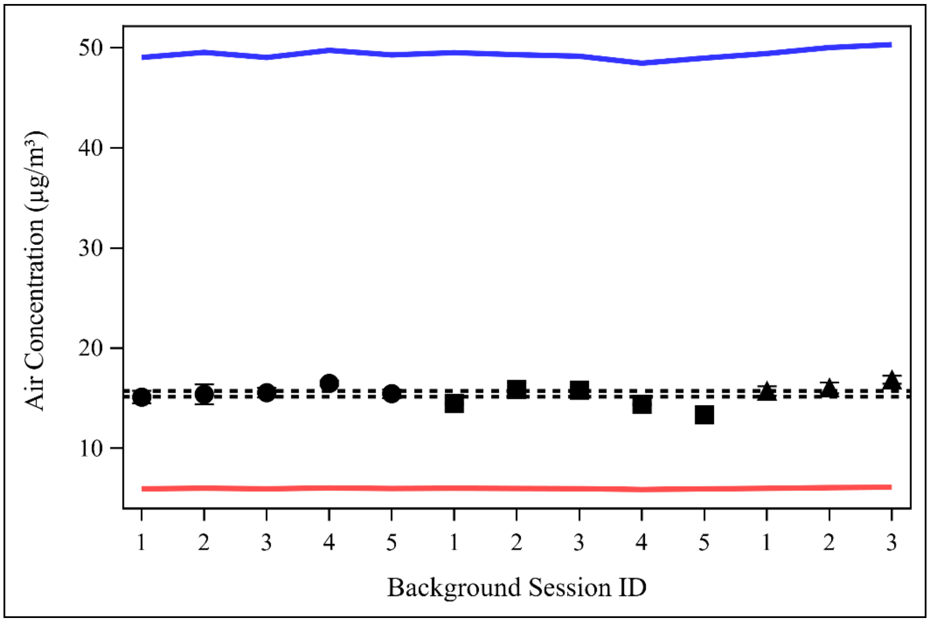

Figure S9. Mean (N=4) background concentrations of Formaldehyde measured prior to and after Group I (solid circles), Group II (solid squares), and Group III (solid triangles) product use at the residential ventilation condition. Error bars represent the standard deviation and maybe obscured by the data point. The 90% Confidence intervals are shown by the dashed lines with the Limit of Detection shown in red and the Limit of Quantification shown in blue.

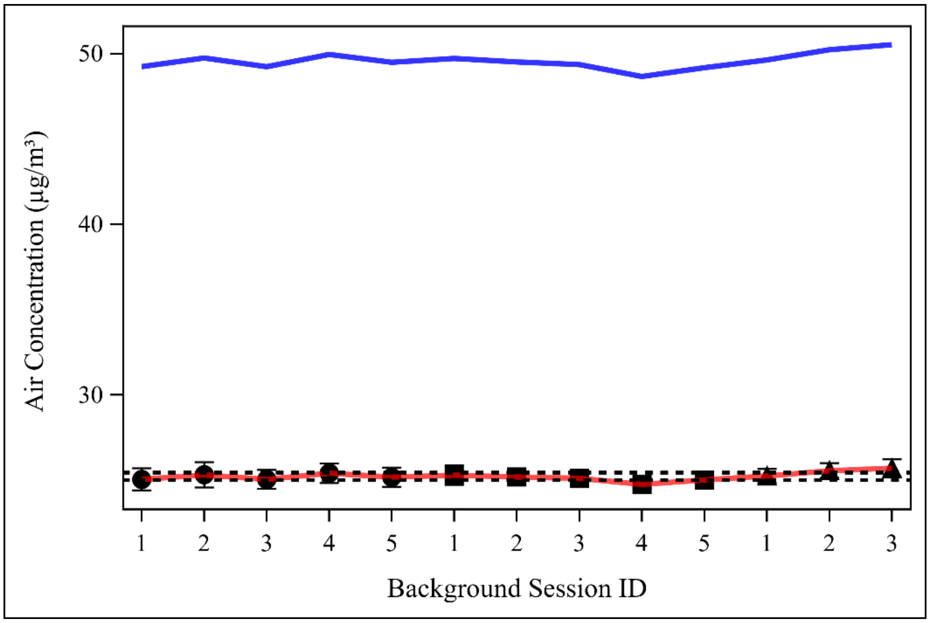

Figure S10. Mean (N=4) background concentrations of Hexaldehyde measured prior to and after Group I (solid circles), Group II (solid squares), and Group III (solid triangles) product use at the residential ventilation condition. Error bars represent the standard deviation and maybe obscured by the data point. The 90% Confidence intervals are shown by the dashed lines with the Limit of Detection shown in red and the Limit of Quantification shown in blue.

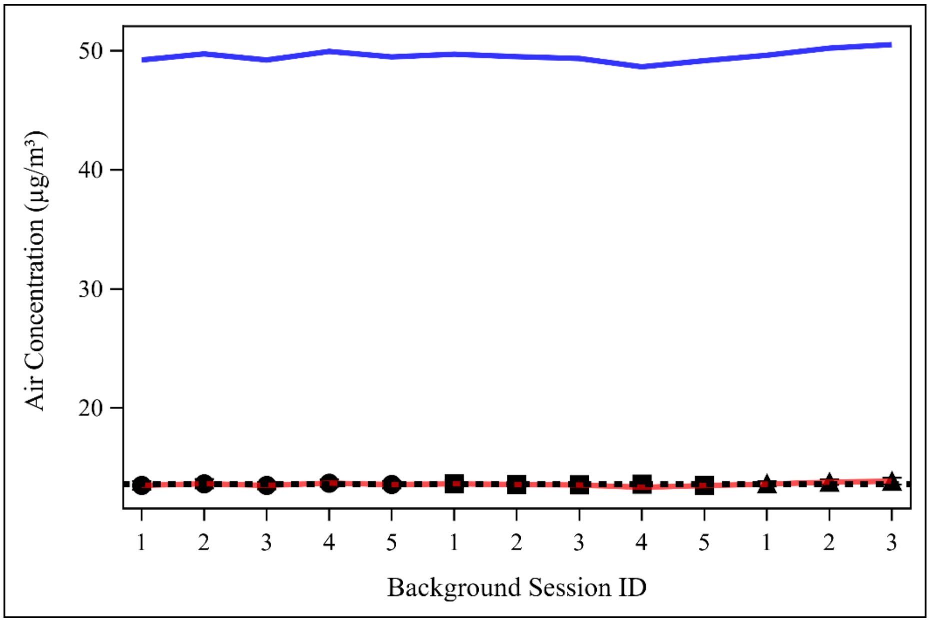

Figure S11. Mean background concentrations of Isovaleraldehyde measured prior to and after Group I (solid circles), Group II (solid squares), and Group III (solid triangles) product use at the residential ventilation condition. Error bars represent the standard deviation and maybe obscured by the data point. The 90% Confidence intervals are shown by the dashed lines with the Limit of Detection shown in red and the Limit of Quantification shown in blue.

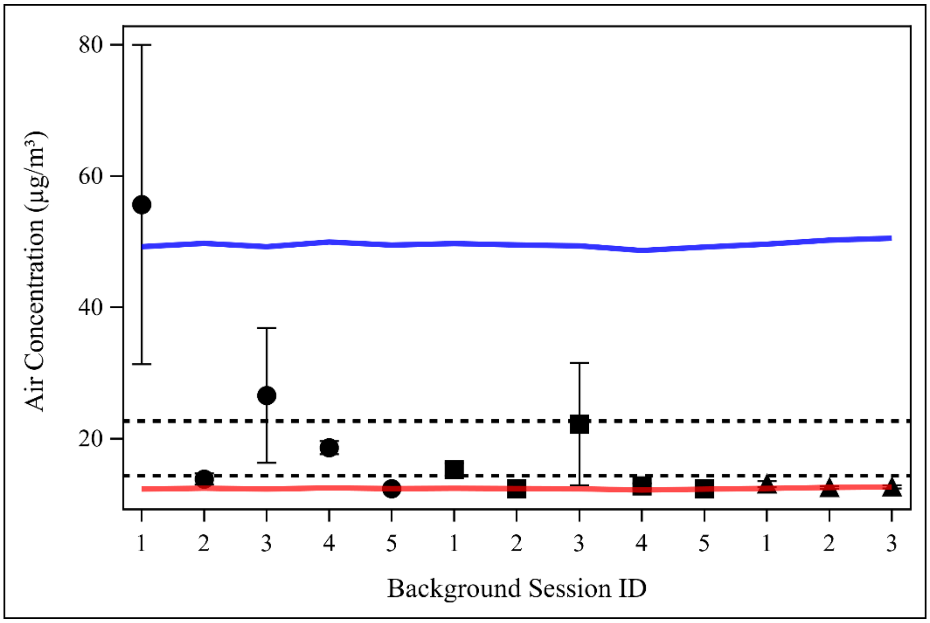

Figure S12. Mean (N=4) background concentrations of Propionaldehyde measured prior to and after Group I (solid circles), Group II (solid squares), and Group III (solid triangles) product use at the residential ventilation condition. Error bars represent the standard deviation and maybe obscured by the data point. The 90% Confidence intervals are shown by the dashed lines with the Limit of Detection shown in red and the Limit of Quantification shown in blue.

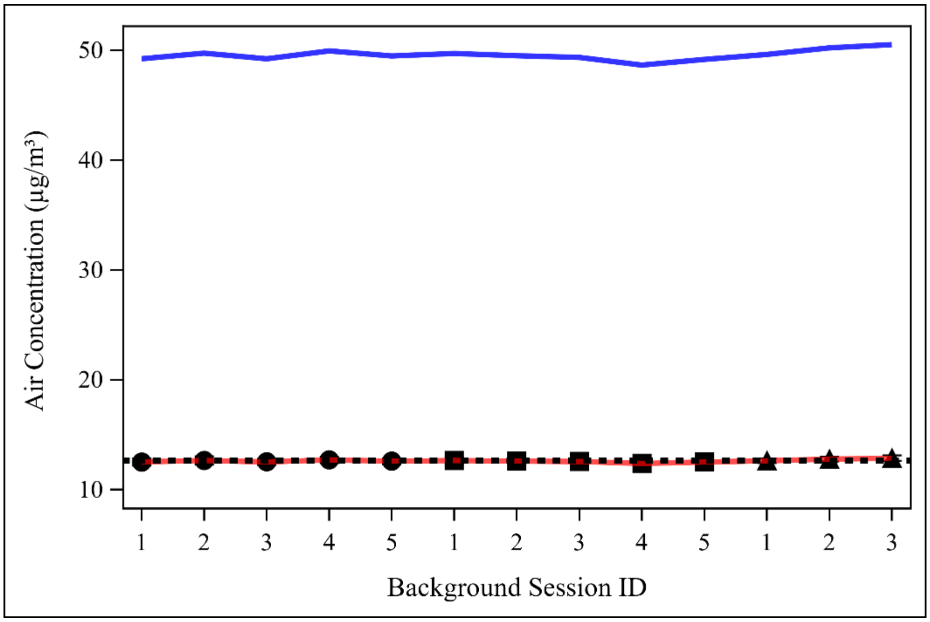

Figure S13. Mean (N=4)background concentrations of Valeraldehyde measured prior to and after Group I (solid circles), Group II (solid squares), and Group III (solid triangles) product use at the residential ventilation condition. Error bars represent the standard deviation and maybe obscured by the data point. The 90% Confidence intervals are shown by the dashed lines with the Limit of Detection shown in red and the Limit of Quantification shown in blue.

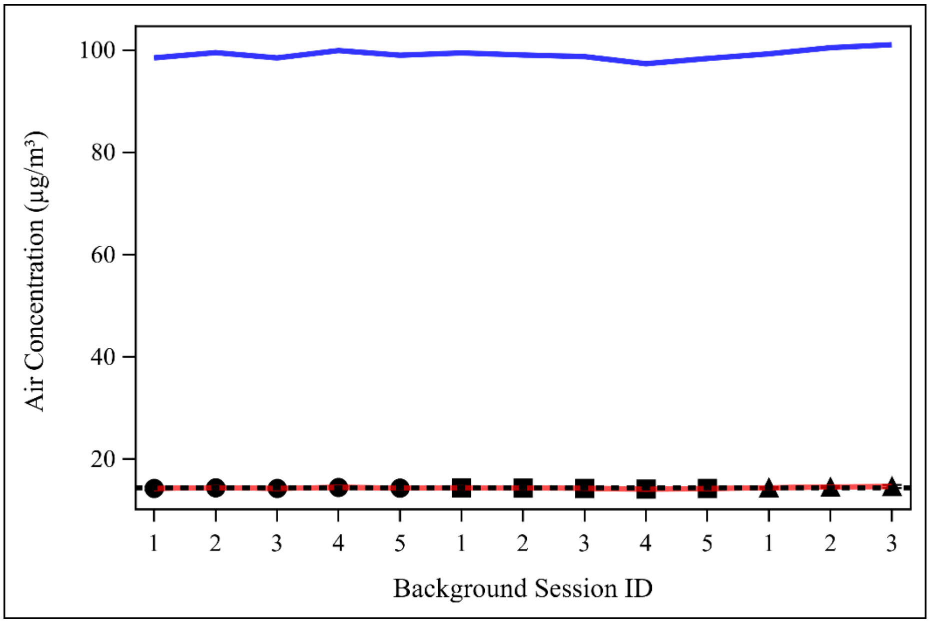

Figure S14. Mean (N=4) background concentrations of m&p-Tolualdehyde measured prior to and after Group I (solid circles), Group II (solid squares), and Group III (solid triangles) product use at the residential ventilation condition. Error bars represent the standard deviation and maybe obscured by the data point. The 90% Confidence intervals are shown by the dashed lines with the Limit of Detection shown in red and the Limit of Quantification shown in blue.

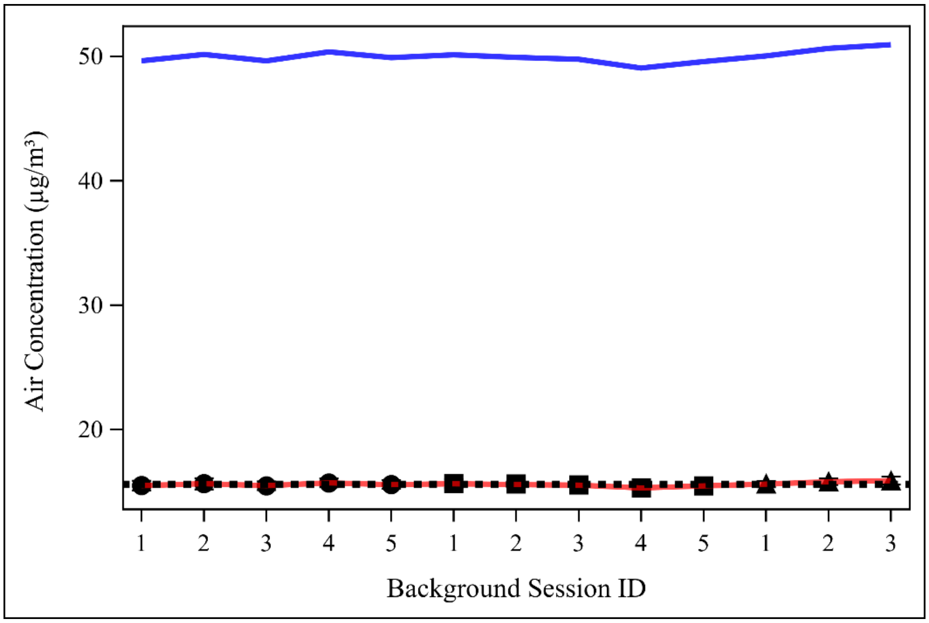

Figure S15. Mean (N=4) background concentrations of Tolualdehyde measured prior to and after Group I (solid circles), Group II (solid squares), and Group III (solid triangles) product use at the residential ventilation condition. Error bars represent the standard deviation and maybe obscured by the data point. The 90% Confidence intervals are shown by the dashed lines with the Limit of Detection shown in red and the Limit of Quantification shown in blue.

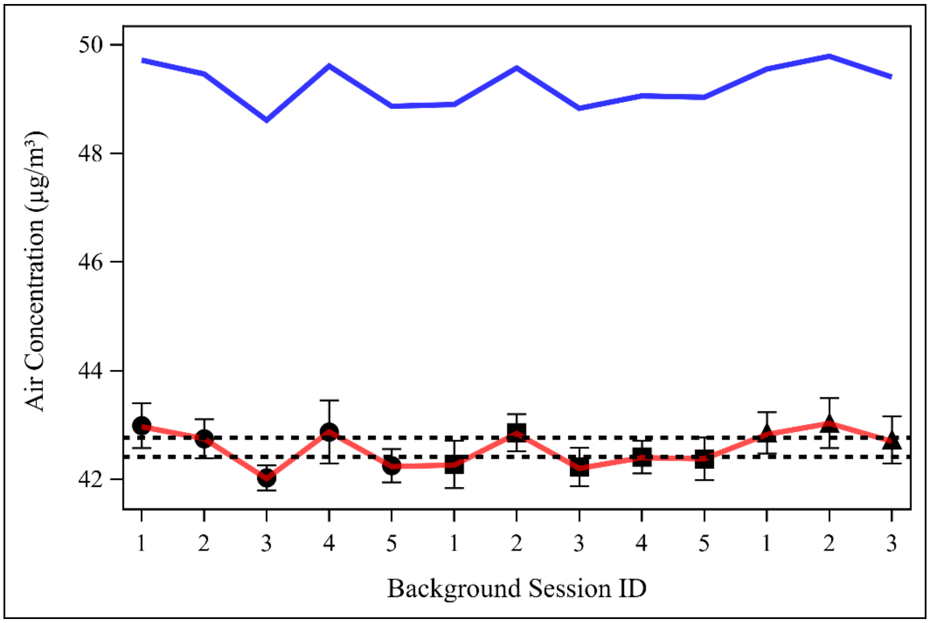

Figure S16. Mean (N=4) background concentrations of 2,5 Dimethylbenzaldehyde measured prior to and after Group I (solid circles), Group II (solid squares), and Group III (solid triangles) product use at the office ventilation condition. Error bars represent the standard deviation and maybe obscured by the data point. The 90% Confidence intervals are shown by the dashed lines with the Limit of Detection shown in red and the Limit of Quantification shown in blue.

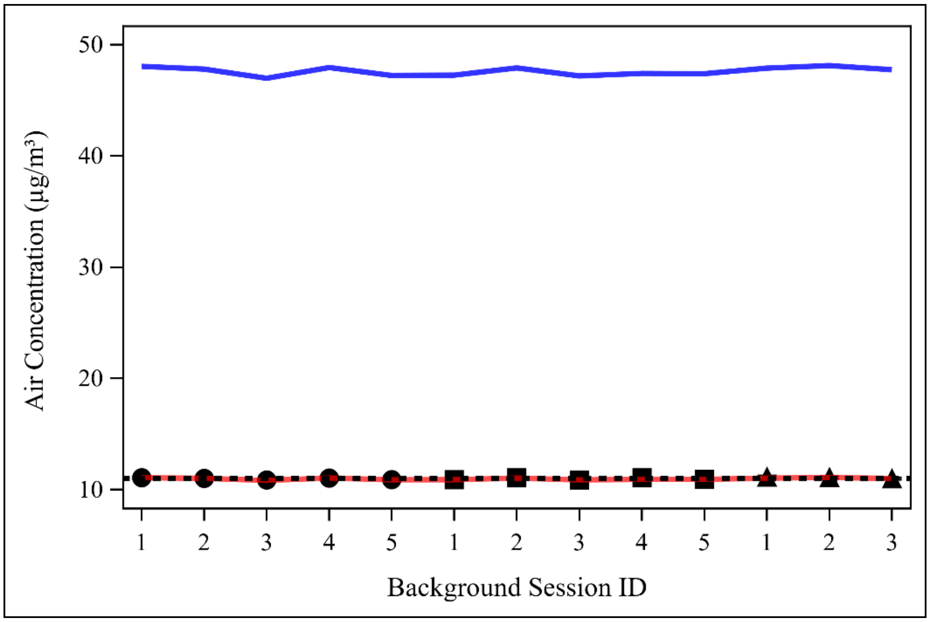

Figure S17. Mean (N=4) background concentrations of 2-Butanone measured prior to and after Group I (solid circles), Group II (solid squares), and Group III (solid triangles) product use at the office ventilation condition. Error bars represent the standard deviation and maybe obscured by the data point. The 90% Confidence intervals are shown by the dashed lines with the Limit of Detection shown in red and the Limit of Quantification shown in blue.

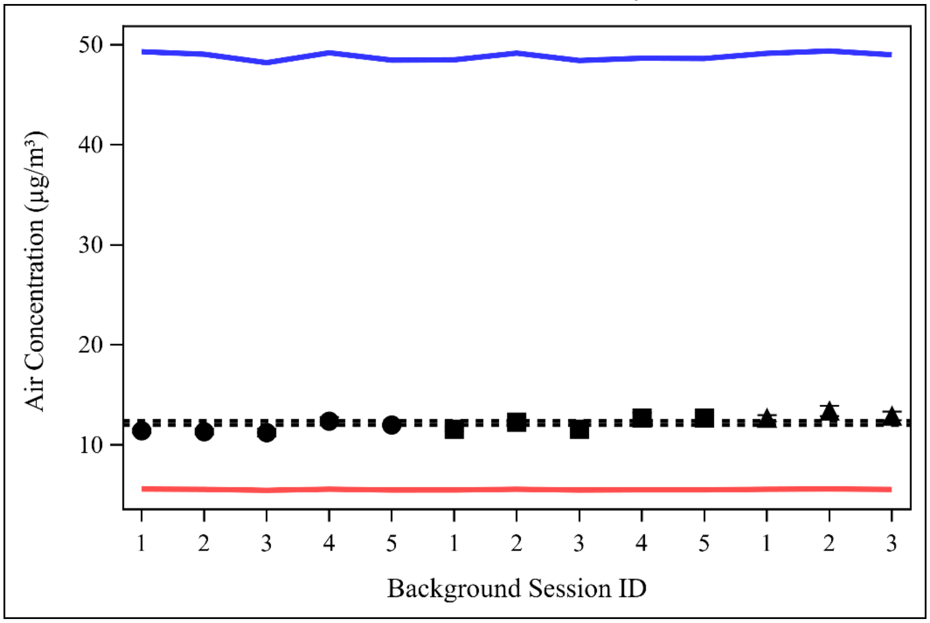

Figure S18. Mean (N=4) background concentrations of Acetaldehyde measured prior to and after Group I (solid circles), Group II (solid squares), and Group III (solid triangles) product use at the office ventilation condition. Error bars represent the standard deviation and maybe obscured by the data point. The 90% Confidence intervals are shown by the dashed lines with the Limit of Detection shown in red and the Limit of Quantification shown in blue.

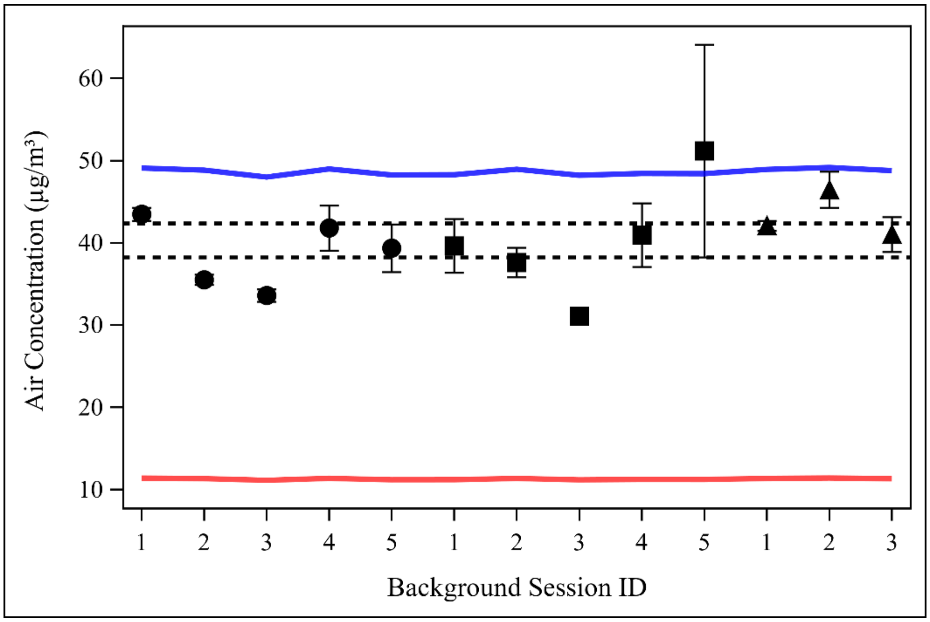

Figure S19. Mean (N=4) background concentrations of Acetone measured prior to and after Group I (solid circles), Group II (solid squares), and Group III (solid triangles) product use at the office ventilation condition. Error bars represent the standard deviation and maybe obscured by the data point. The 90% Confidence intervals are shown by the dashed lines with the Limit of Detection shown in red and the Limit of Quantification shown in blue.

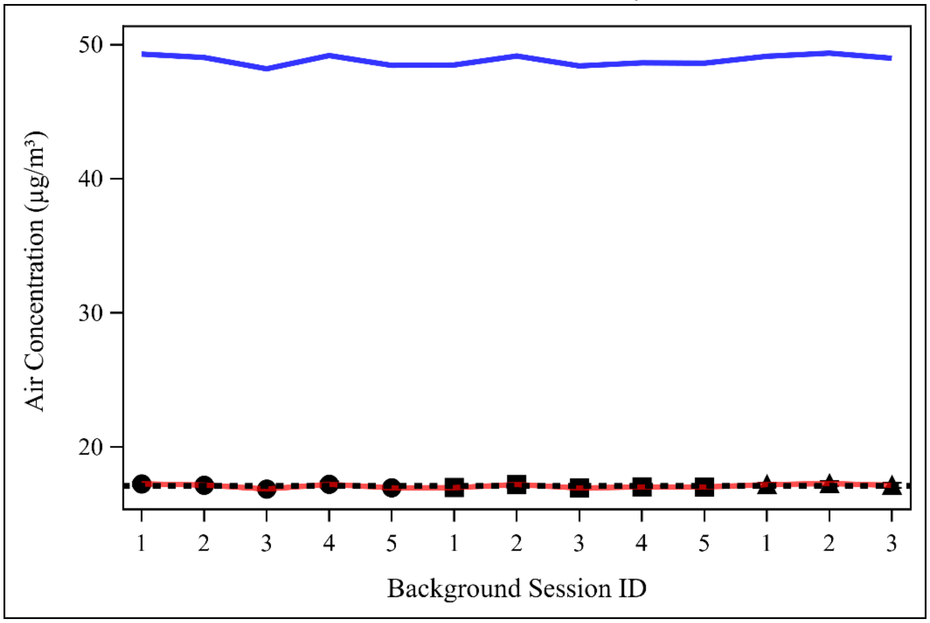

Figure S20. Mean (N=4) background concentrations of Acrolein measured prior to and after Group I (solid circles), Group II (solid squares), and Group III (solid triangles) product use at the office ventilation condition. Error bars represent the standard deviation and maybe obscured by the data point. The 90% Confidence intervals are shown by the dashed lines with the Limit of Detection shown in red and the Limit of Quantification shown in blue.

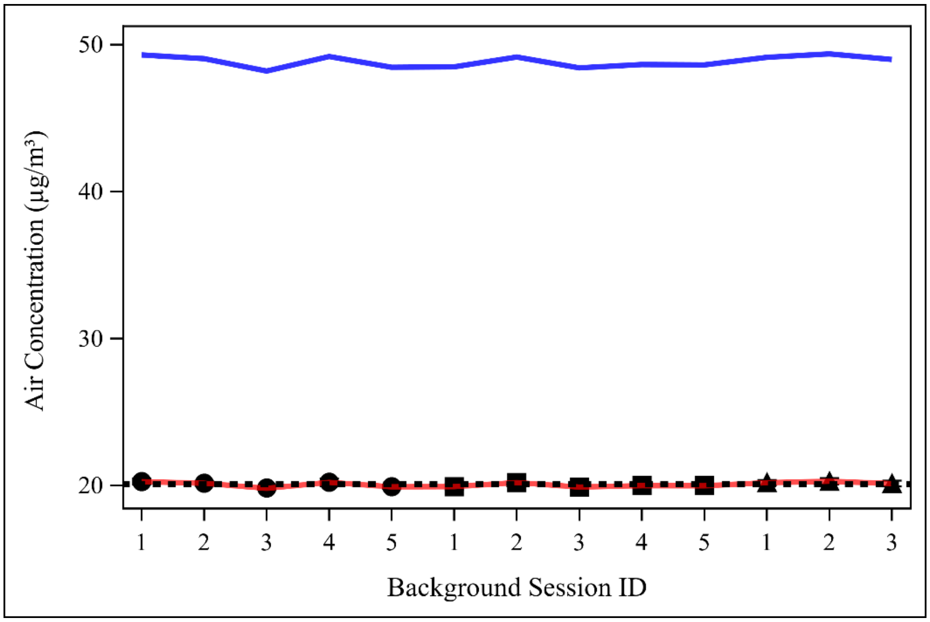

Figure S21. Mean (N=4) background concentrations of Benzaldehyde measured prior to and after Group I (solid circles), Group II (solid squares), and Group III (solid triangles) product use at the office ventilation condition. Error bars represent the standard deviation and maybe obscured by the data point. The 90% Confidence intervals are shown by the dashed lines with the Limit of Detection shown in red and the Limit of Quantification shown in blue.

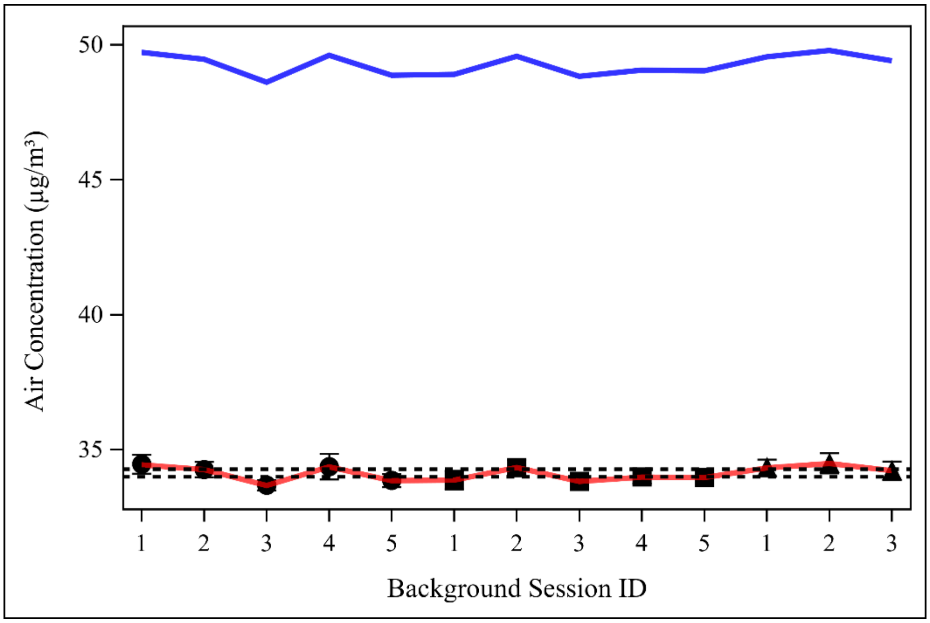

Figure S22. Mean (N=4) background concentrations of Butyraldehyde measured prior to and after Group I (solid circles), Group II (solid squares), and Group III (solid triangles) product use at the office ventilation condition. Error bars represent the standard deviation and maybe obscured by the data point. The 90% Confidence intervals are shown by the dashed lines with the Limit of Detection shown in red and the Limit of Quantification shown in blue.

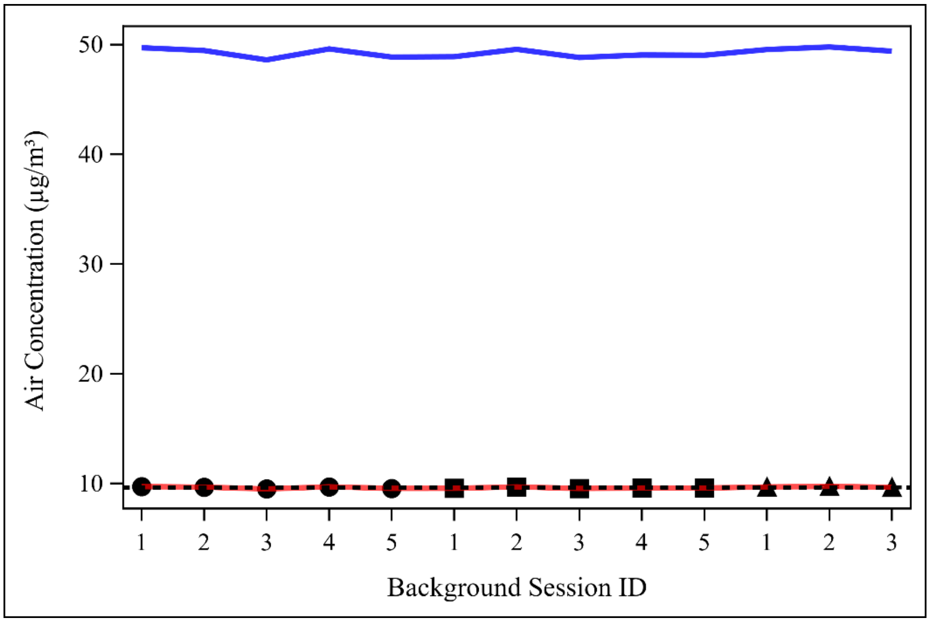

Figure S23. Mean (N=4) background concentrations of Crotonaldehyde measured prior to and after Group I (solid circles), Group II (solid squares), and Group III (solid triangles) product use at the office ventilation condition. Error bars represent the standard deviation and maybe obscured by the data point. The 90% Confidence intervals are shown by the dashed lines with the Limit of Detection shown in red and the Limit of Quantification shown in blue.

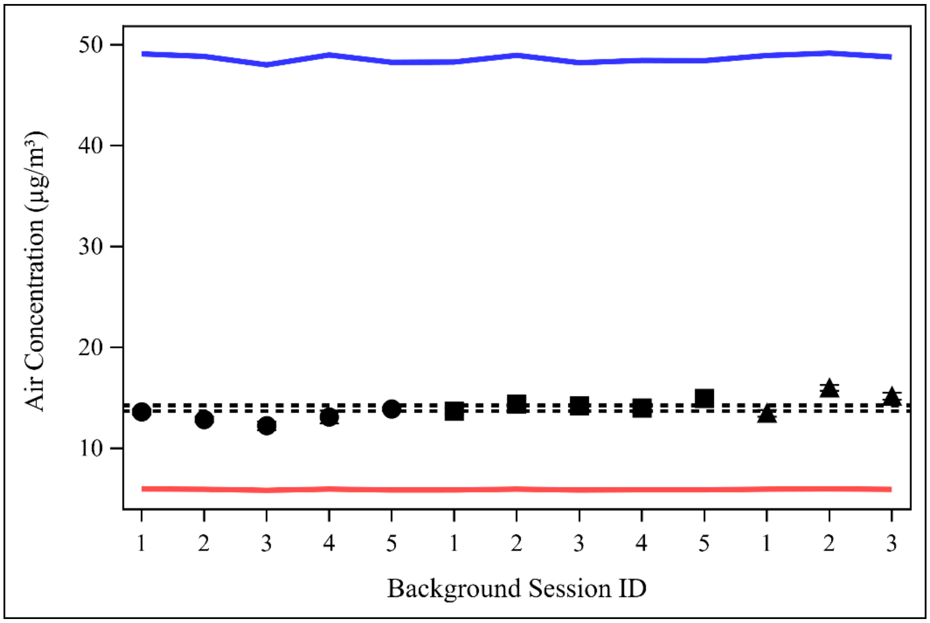

Figure S24. Mean (N=4) background concentrations of Formaldehyde measured prior to and after Group I (solid circles), Group II (solid squares), and Group III (solid triangles) product use at the office ventilation condition. Error bars represent the standard deviation and maybe obscured by the data point. The 90% Confidence intervals are shown by the dashed lines with the Limit of Detection shown in red and the Limit of Quantification shown in blue.

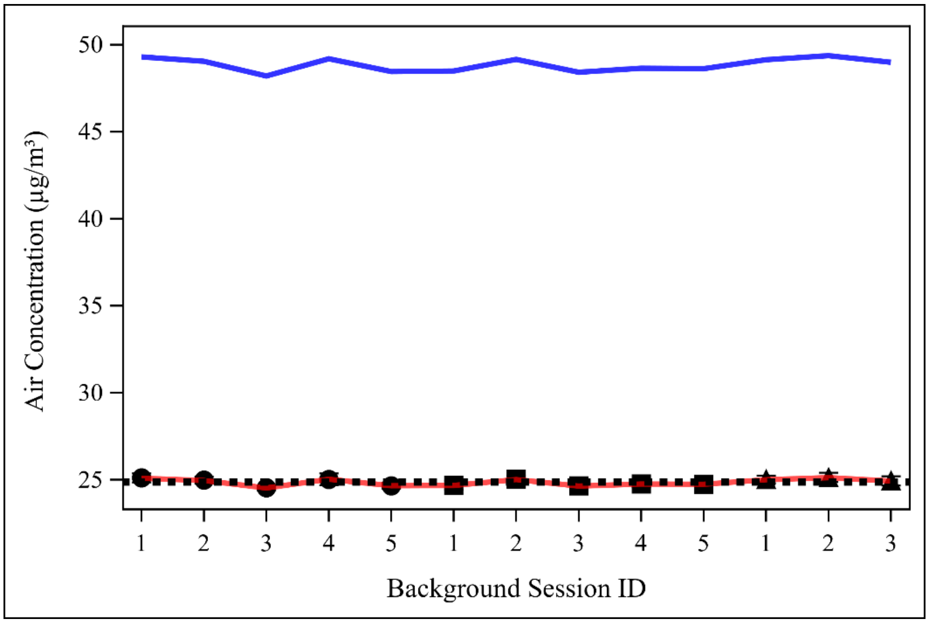

Figure S25. Mean (N=4) background concentrations of Hexaldehyde measured prior to and after Group I (solid circles), Group II (solid squares), and Group III (solid triangles) product use at the office ventilation condition. Error bars represent the standard deviation and maybe obscured by the data point. The 90% Confidence intervals are shown by the dashed lines with the Limit of Detection shown in red and the Limit of Quantification shown in blue.

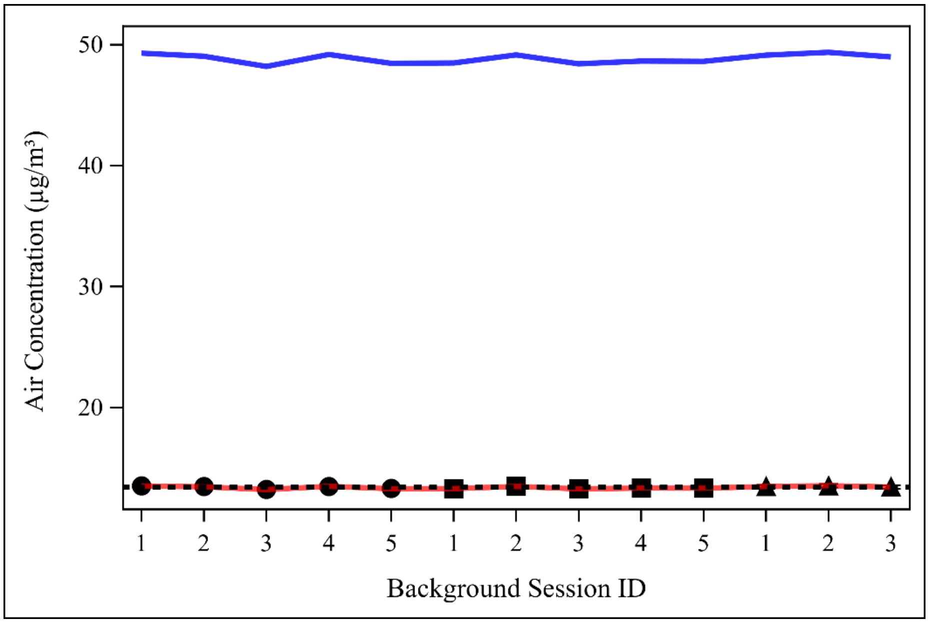

Figure S26. Mean (N=4) background concentrations of Isovaleraldehyde measured prior to and after Group I (solid circles), Group II (solid squares), and Group III (solid triangles) product use at the office ventilation condition. Error bars represent the standard deviation and maybe obscured by the data point. The 90% Confidence intervals are shown by the dashed lines with the Limit of Detection shown in red and the Limit of Quantification shown in blue.

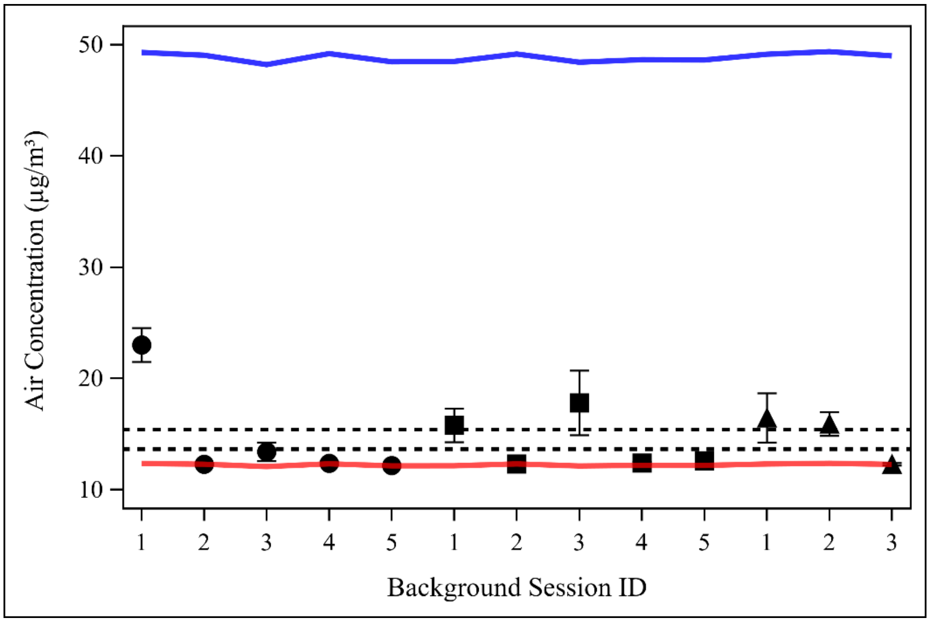

Figure S27. Mean (N=4) background concentrations of Propionaldehyde measured prior to and after Group I (solid circles), Group II (solid squares), and Group III (solid triangles) product use at the office ventilation condition. Error bars represent the standard deviation and maybe obscured by the data point. The 90% Confidence intervals are shown by the dashed lines with the Limit of Detection shown in red and the Limit of Quantification shown in blue.

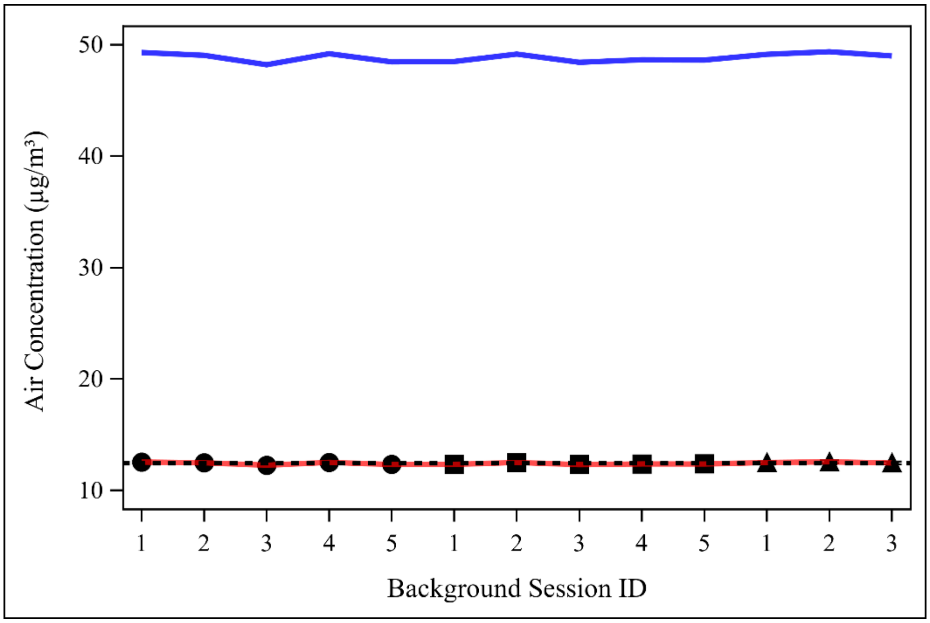

Figure S28. Mean (N=4) background concentrations of Valeraldehyde measured prior to and after Group I (solid circles), Group II (solid squares), and Group III (solid triangles) product use at the office ventilation condition. Error bars represent the standard deviation and maybe obscured by the data point. The 90% Confidence intervals are shown by the dashed lines with the Limit of Detection shown in red and the Limit of Quantification shown in blue.

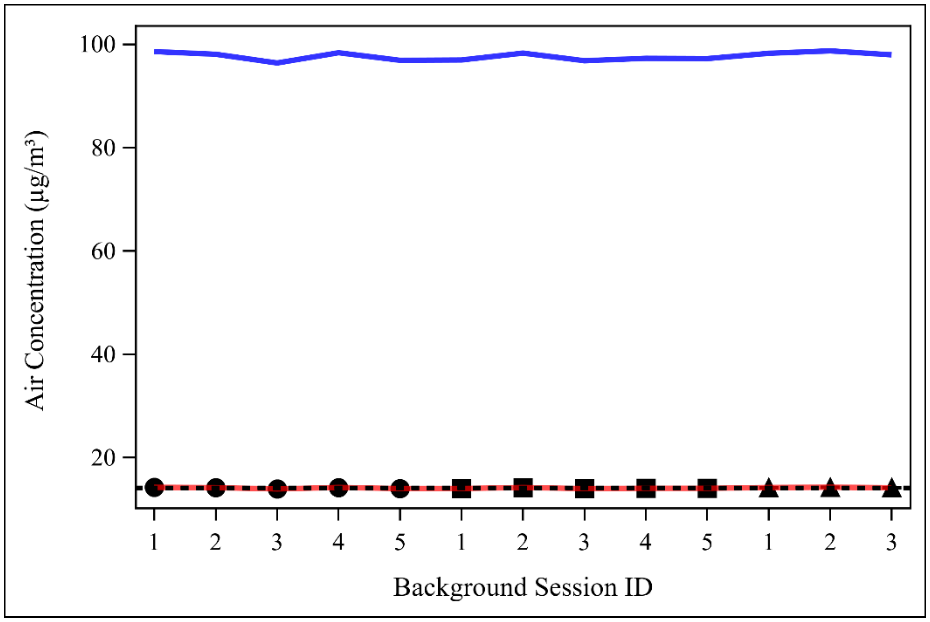

Figure S29. Mean (N=4) background concentrations of m&p-Tolualdehyde measured prior to and after Group I (solid circles), Group II (solid squares), and Group III (solid triangles) product use at the office ventilation condition. Error bars represent the standard deviation and maybe obscured by the data point. The 90% Confidence intervals are shown by the dashed lines with the Limit of Detection shown in red and the Limit of Quantification shown in blue.

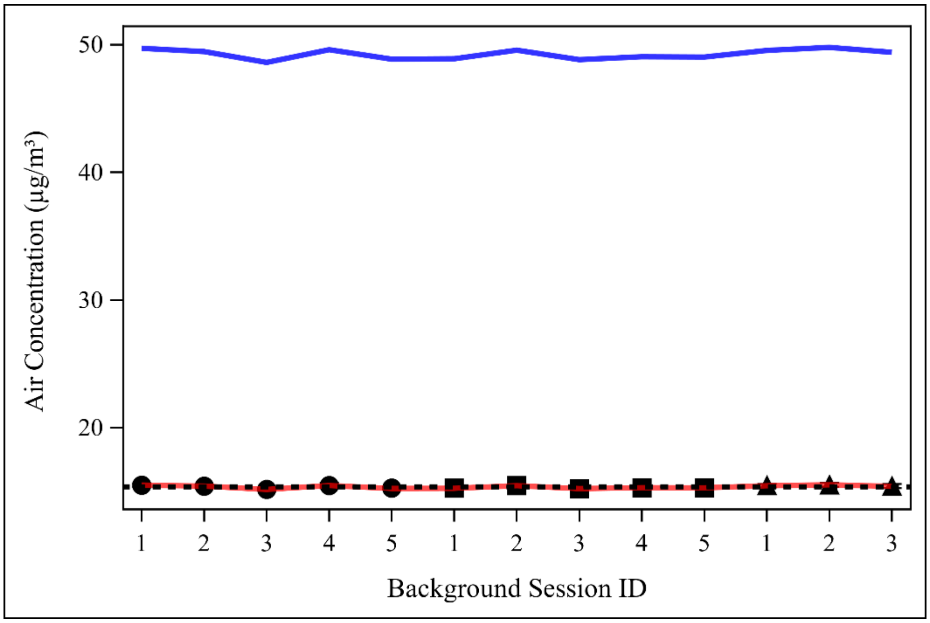

Figure S30. Mean (N=4) background concentrations of Tolualdehyde measured prior to and after Group I (solid circles), Group II (solid squares), and Group III (solid triangles) product use at the office ventilation condition. Error bars represent the standard deviation and maybe obscured by the data point. The 90% Confidence intervals are shown by the dashed lines with the Limit of Detection shown in red and the Limit of Quantification shown in blue.

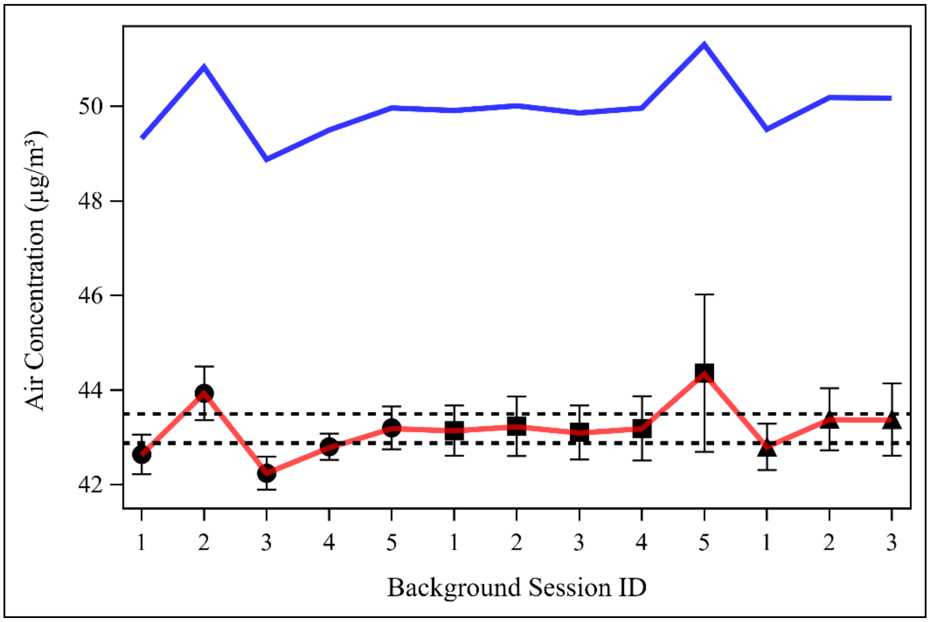

Figure S31. Mean (N=4) background concentrations of 2,5 Dimethylbenzaldehyde measured prior to and after Group I (solid circles), Group II (solid squares), and Group III (solid triangles) product use at the hospitality ventilation condition. Error bars represent the standard deviation and maybe obscured by the data point. The 90% Confidence intervals are shown by the dashed lines with the Limit of Detection shown in red and the Limit of Quantification shown in blue.

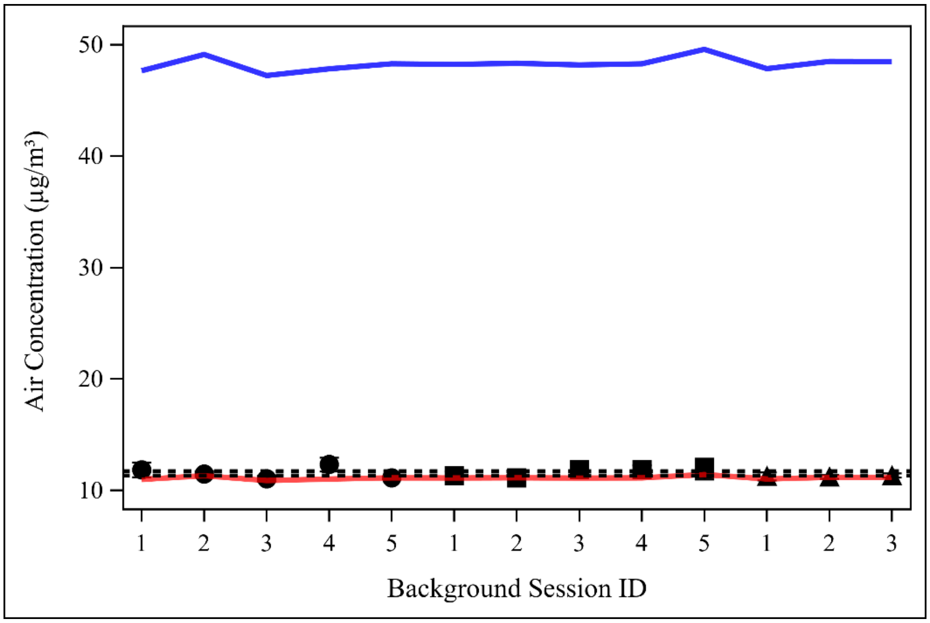

Figure S32. Mean (N=4) background concentrations of 2-Butanone measured prior to and after Group I (solid circles), Group II (solid squares), and Group III (solid triangles) product use at the hospitality ventilation condition. Error bars represent the standard deviation and maybe obscured by the data point. The 90% Confidence intervals are shown by the dashed lines with the Limit of Detection shown in red and the Limit of Quantification shown in blue.

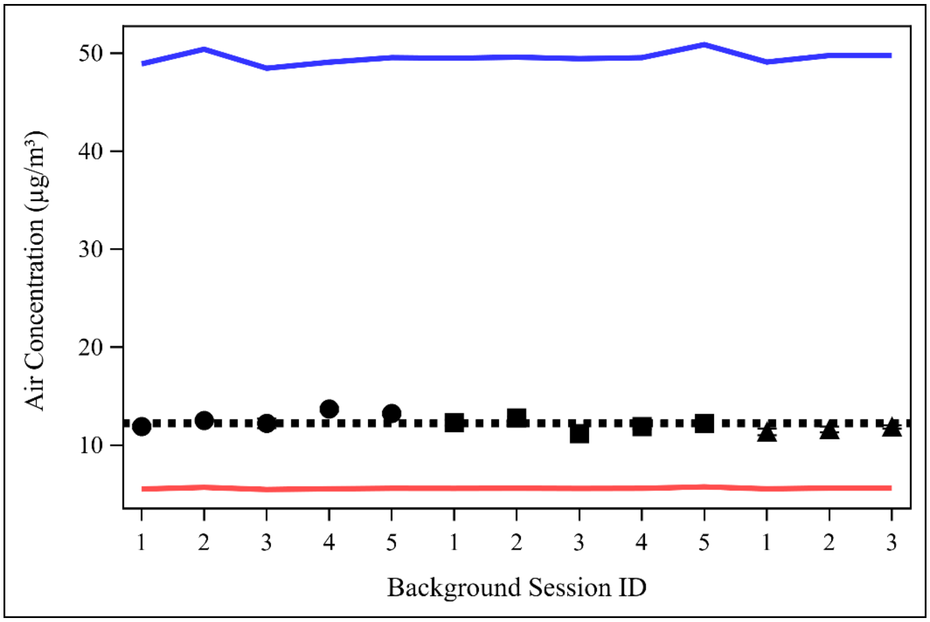

Figure S33. Mean (N=4) background concentrations of Acetaldehyde measured prior to and after Group I (solid circles), Group II (solid squares), and Group III (solid triangles) product use at the hospitality ventilation condition. Error bars represent the standard deviation and maybe obscured by the data point. The 90% Confidence intervals are shown by the dashed lines with the Limit of Detection shown in red and the Limit of Quantification shown in blue.

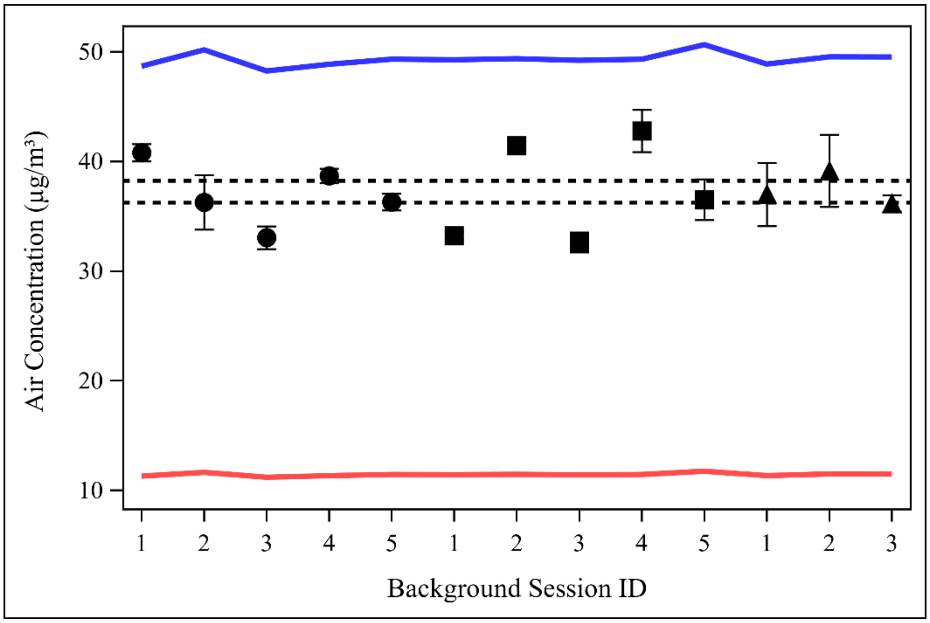

Figure S34. Mean (N=4) background concentrations of Acetone measured prior to and after Group I (solid circles), Group II (solid squares), and Group III (solid triangles) product use at the hospitality ventilation condition. Error bars represent the standard deviation and maybe obscured by the data point. The 90% Confidence intervals are shown by the dashed lines with the Limit of Detection shown in red and the Limit of Quantification shown in blue.

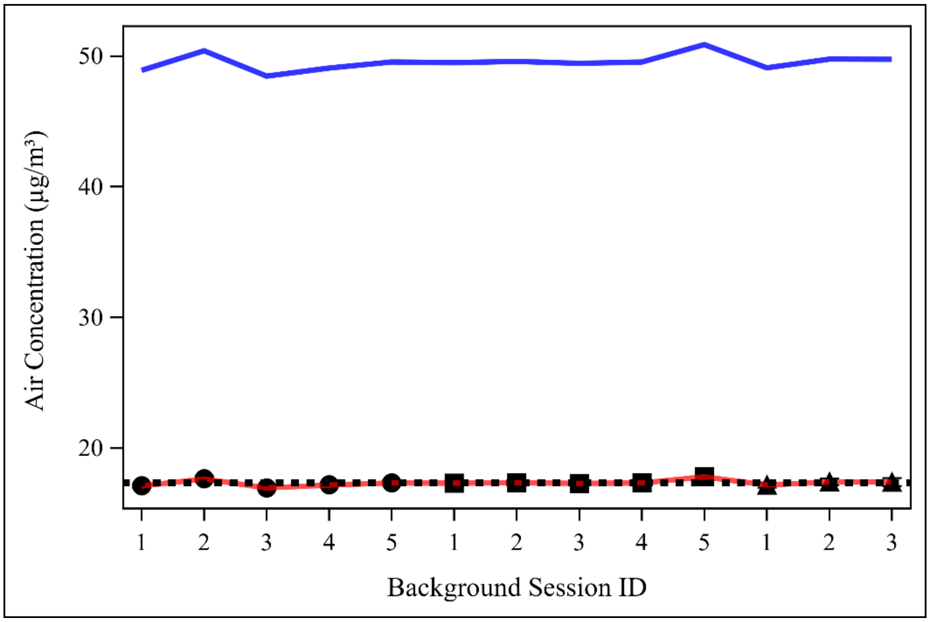

Figure S35. Mean (N=4) background concentrations of Acrolein measured prior to and after Group I (solid circles), Group II (solid squares), and Group III (solid triangles) product use at the hospitality ventilation condition. Error bars represent the standard deviation and maybe obscured by the data point. The 90% Confidence intervals are shown by the dashed lines with the Limit of Detection shown in red and the Limit of Quantification shown in blue.

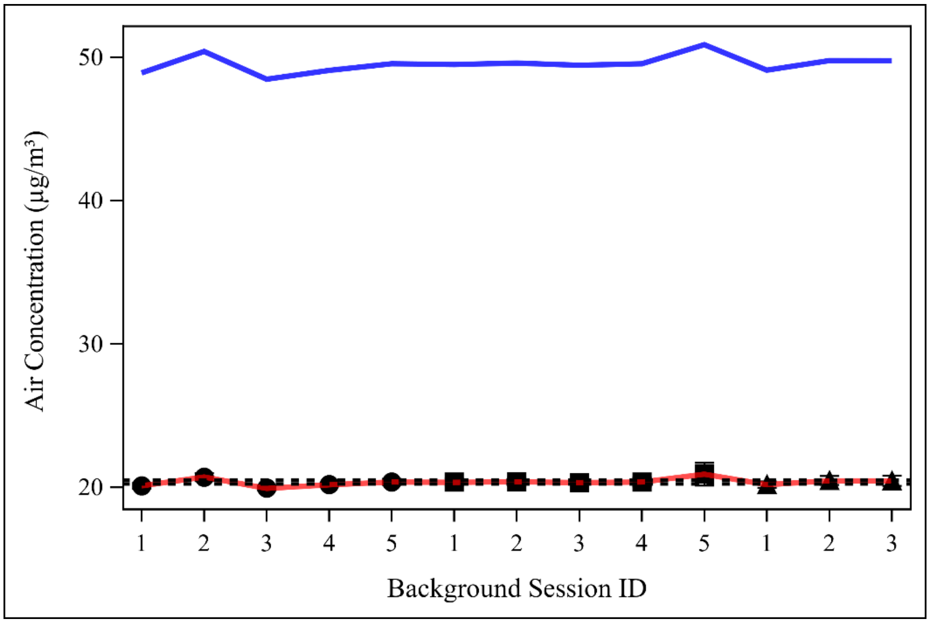

Figure S36. Mean (N=4) background concentrations of Benzaldehyde measured prior to and after Group I (solid circles), Group II (solid squares), and Group III (solid triangles) product use at the hospitality ventilation condition. Error bars represent the standard deviation and maybe obscured by the data point. The 90% Confidence intervals are shown by the dashed lines with the Limit of Detection shown in red and the Limit of Quantification shown in blue.

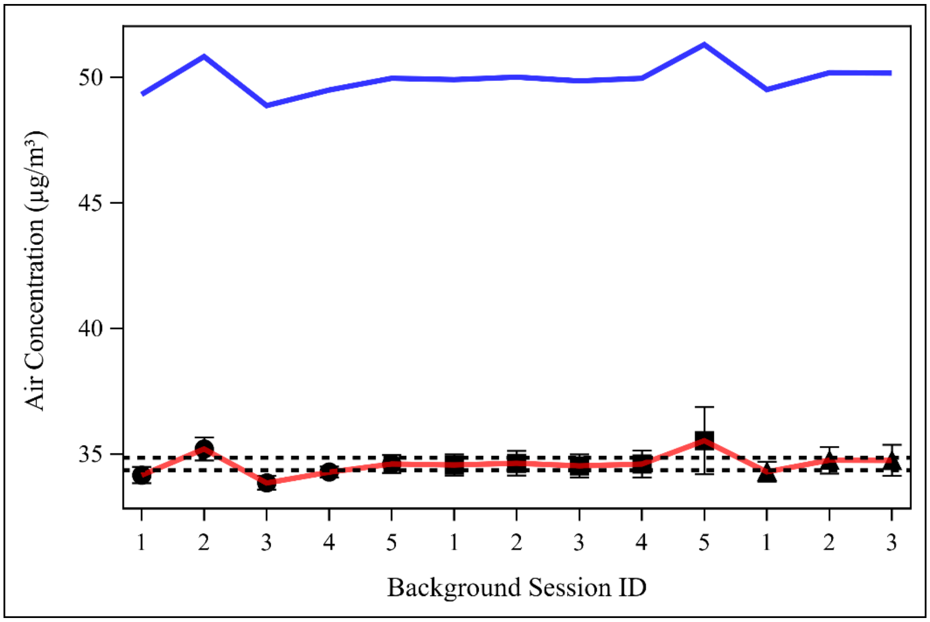

Figure S37. Mean (N=4) background concentrations of Butyraldehyde measured prior to and after Group I (solid circles), Group II (solid squares), and Group III (solid triangles) product use at the hospitality ventilation condition. Error bars represent the standard deviation and maybe obscured by the data point. The 90% Confidence intervals are shown by the dashed lines with the Limit of Detection shown in red and the Limit of Quantification shown in blue.

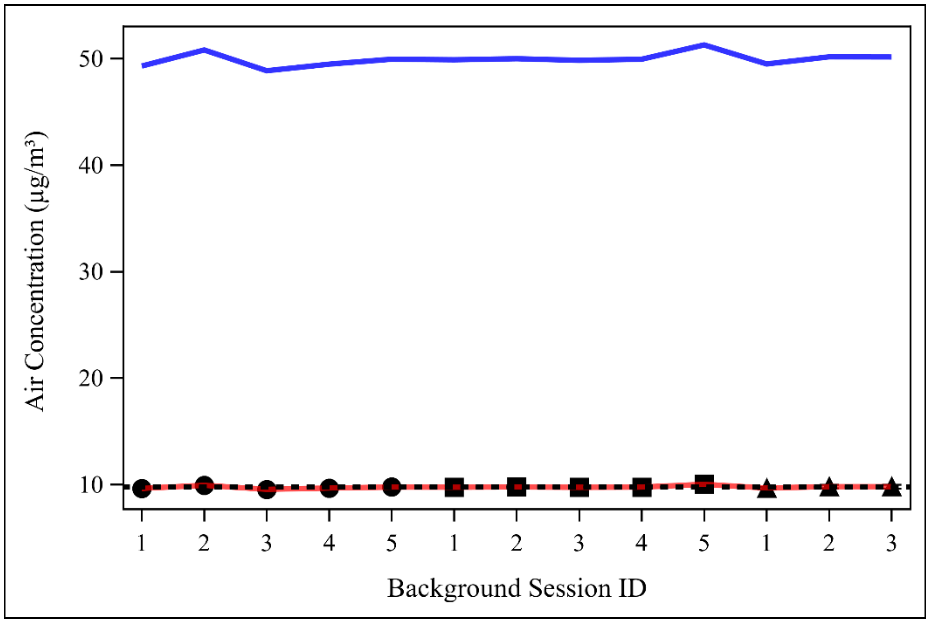

Figure S38. Mean (N=4) background concentrations of Crotonaldehyde measured prior to and after Group I (solid circles), Group II (solid squares), and Group III (solid triangles) product use at the hospitality ventilation condition. Error bars represent the standard deviation and maybe obscured by the data point. The 90% Confidence intervals are shown by the dashed lines with the Limit of Detection shown in red and the Limit of Quantification shown in blue.

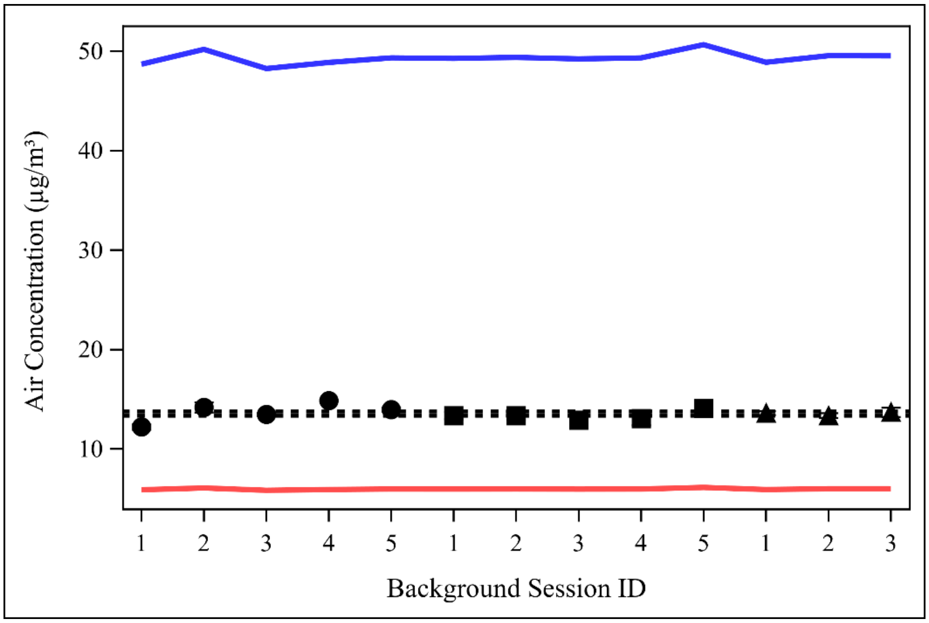

Figure S39. Mean (N=4) background concentrations of Formaldehyde measured prior to and after Group I (solid circles), Group II (solid squares), and Group III (solid triangles) product use at the hospitality ventilation condition. Error bars represent the standard deviation and maybe obscured by the data point. The 90% Confidence intervals are shown by the dashed lines with the Limit of Detection shown in red and the Limit of Quantification shown in blue.

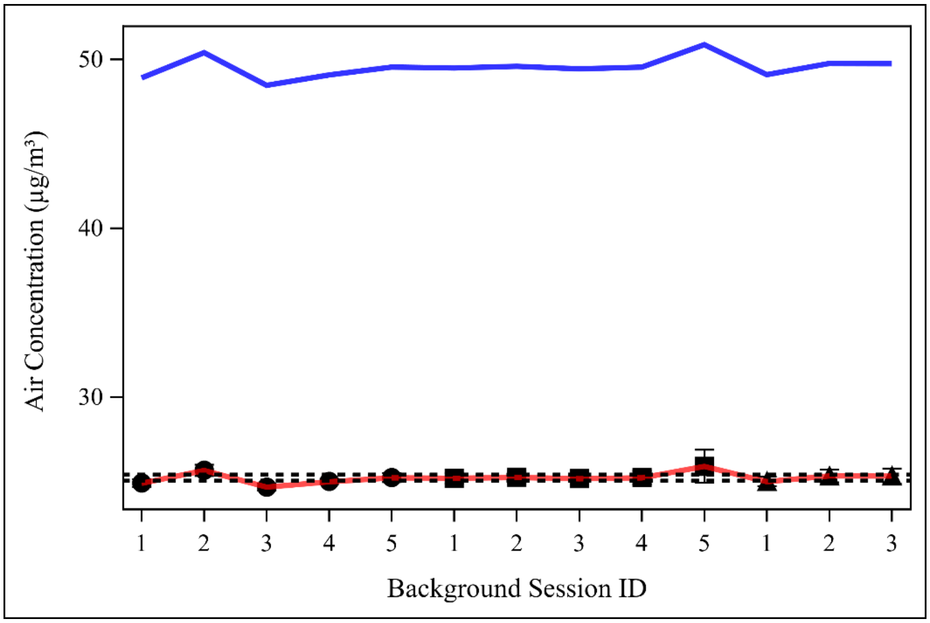

Figure S40. Mean (N=4) background concentrations of Hexaldehyde measured prior to and after Group I (solid circles), Group II (solid squares), and Group III (solid triangles) product use at the hospitality ventilation condition. Error bars represent the standard deviation and maybe obscured by the data point. The 90% Confidence intervals are shown by the dashed lines with the Limit of Detection shown in red and the Limit of Quantification shown in blue.

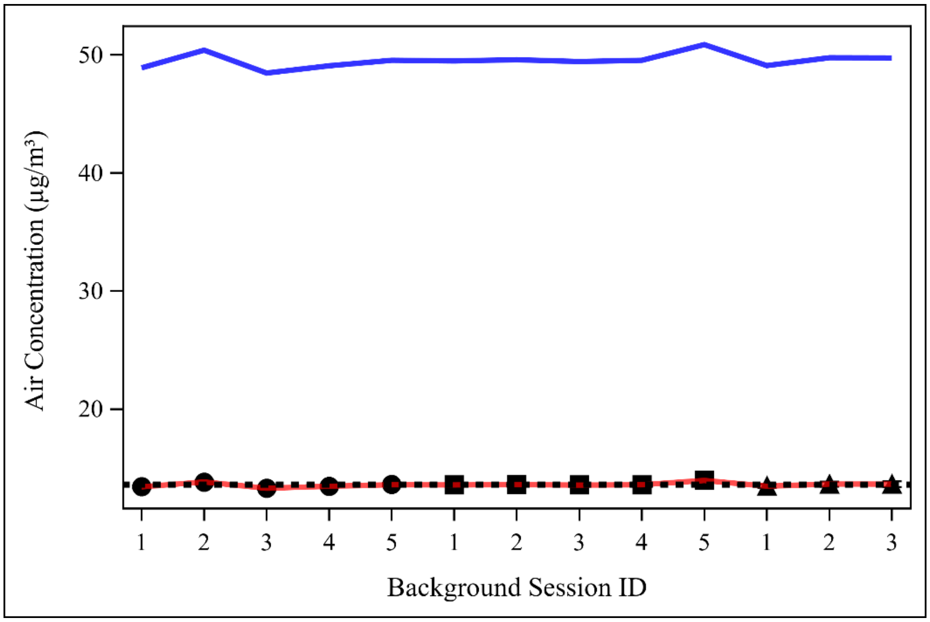

Figure S41. Mean (N=4) background concentrations of Isovaleraldehyde measured prior to and after Group I (solid circles), Group II (solid squares), and Group III (solid triangles) product use at the hospitality ventilation condition. Error bars represent the standard deviation and maybe obscured by the data point. The 90% Confidence intervals are shown by the dashed lines with the Limit of Detection shown in red and the Limit of Quantification shown in blue.

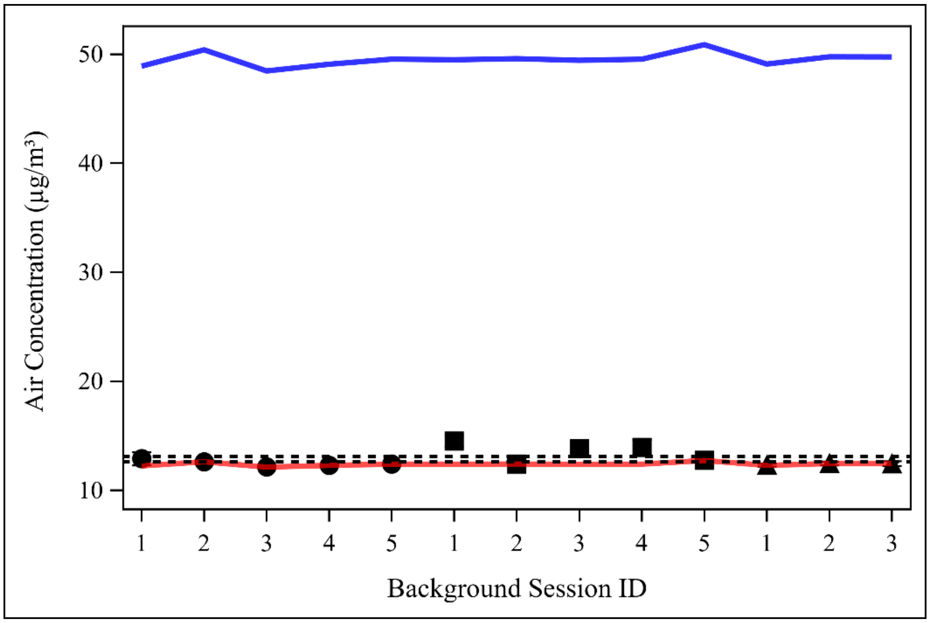

Figure S42. Mean (N=4) background concentrations of Propionaldehyde measured prior to and after Group I (solid circles), Group II (solid squares), and Group III (solid triangles) product use at the hospitality ventilation condition. Error bars represent the standard deviation and maybe obscured by the data point. The 90% Confidence intervals are shown by the dashed lines with the Limit of Detection shown in red and the Limit of Quantification shown in blue.

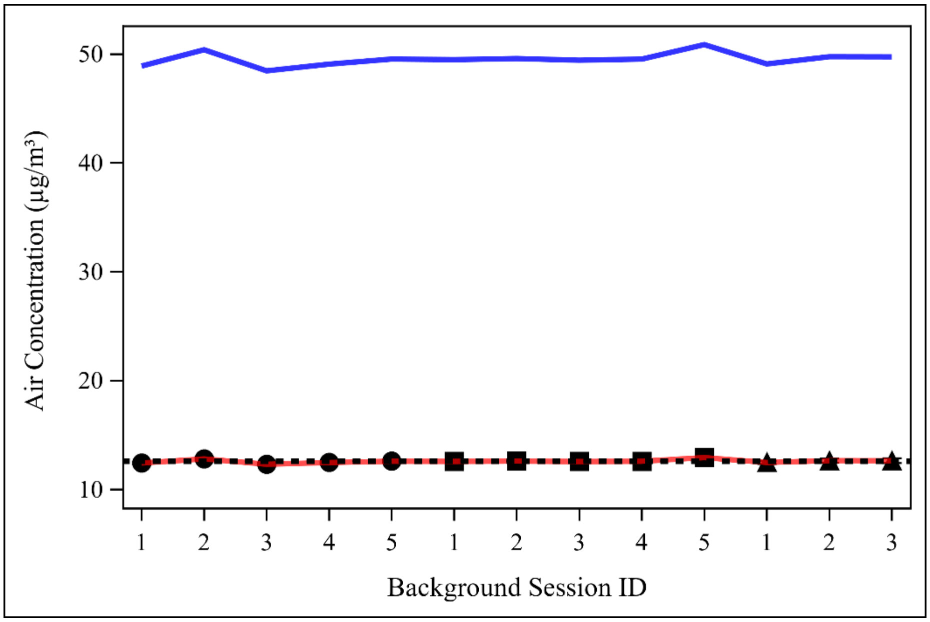

Figure S43. Mean (N=4) background concentrations of Valeraldehyde measured prior to and after Group I (solid circles), Group II (solid squares), and Group III (solid triangles) product use at the hospitality ventilation condition. Error bars represent the standard deviation and maybe obscured by the data point. The 90% Confidence intervals are shown by the dashed lines with the Limit of Detection shown in red and the Limit of Quantification shown in blue.

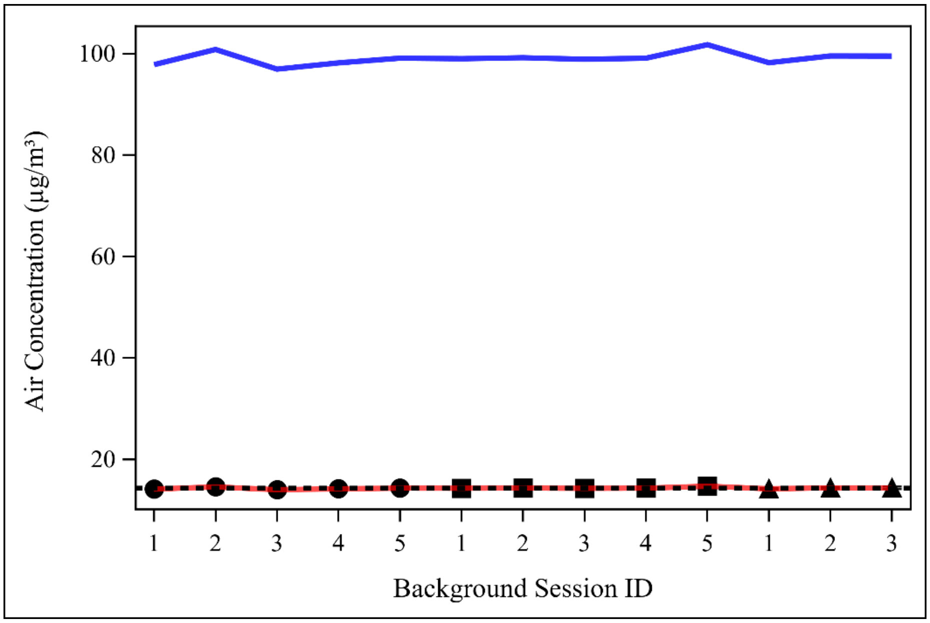

Figure S44. Mean (N=4) background concentrations of m&p-Tolualdehyde measured prior to and after Group I (solid circles), Group II (solid squares), and Group III (solid triangles) product use at the hospitality ventilation condition. Error bars represent the standard deviation and maybe obscured by the data point. The 90% Confidence intervals are shown by the dashed lines with the Limit of Detection shown in red and the Limit of Quantification shown in blue.

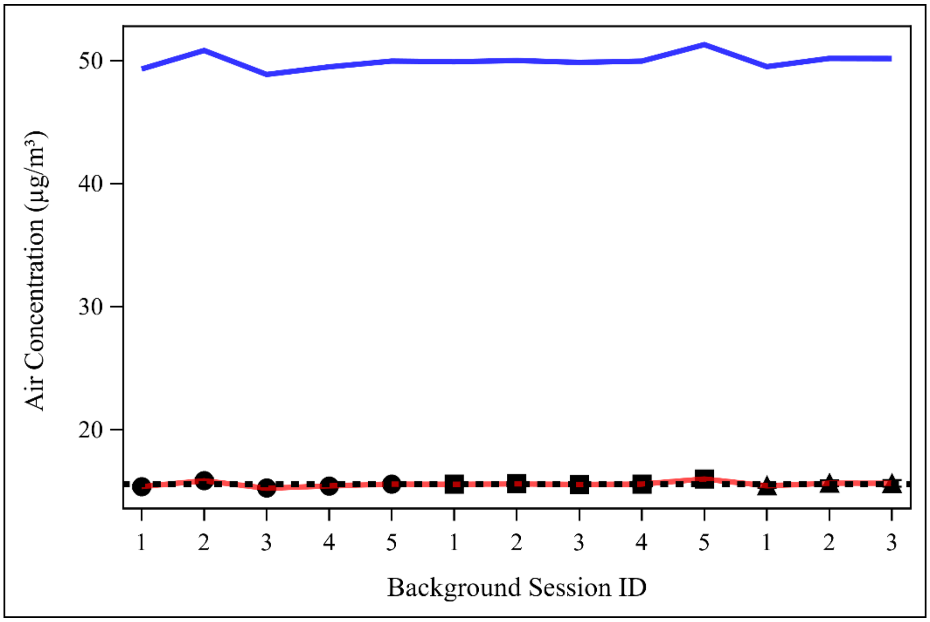

Figure S45. Mean (N=4) background concentrations of Tolualdehyde measured prior to and after Group I (solid circles), Group II (solid squares), and Group III (solid triangles) product use at the hospitality ventilation condition. Error bars represent the standard deviation and maybe obscured by the data point. The 90% Confidence intervals are shown by the dashed lines with the Limit of Detection shown in red and the Limit of Quantification shown in blue.
